# Harnessing nutrient scarcity for enhanced CAR-T-cell potency and safety in solid tumors

**DOI:** 10.1038/s41423-025-01290-x

**Published:** 2025-05-08

**Authors:** Enzo Manchon, Nell Hirt, Benjamin Versier, Aravindhan Soundiramourty, Ludmila Juricek, Celeste Lebbe, Maxime Battistella, Yves Christen, Jacques Mallet, Dominique Charron, Nabila Jabrane-Ferrat, Che Serguera, Reem Al-Daccak

**Affiliations:** 1 National Institute of Health and Medical Research (INSERM) UMRS-976 HIPI, Paris University, Saint-Louis Hospital, 75010 Paris, France; 2 Asfalia Biologics, ICM, Hôpital Pitié-Salpêtrière, 75013 Paris, France; 3 Dermato-Oncology and CIC AP-HP Hôpital Saint Louis, Cancer Institute APHP, Nord-Université Paris Cité, 75010 Paris, France; 4 Department of Pathology, Saint-Louis University Hospital, AP-HP, 75010 Paris, France; 5 Institute for Infectious and Inflammatory Diseases, CNRS UMR5051, INSERM UMR1291, University of Toulouse, 31059 Toulouse, France; 6 Sorbonne Université, Institut du Cerveau - Paris Brain Institute - ICM, Inserm, CNRS, 75013 Paris, France; 7 Present Address: Coave Therapeutics, ICM, Hôpital Pitié-Salpêtrière, Paris, France

**Keywords:** CAR-T, Solid tumours, Amino acid scarcity, Tumour microenvironment, Immunotherapy, Cancer

## Abstract

Despite significant advancements, the effectiveness of chimeric antigen receptor (CAR)-T-cell-based therapies in solid tumors remains limited. Key challenges include on-target effects, off-tumor toxicity and reduced CAR-T-cell function within the tumor microenvironment, which is often characterized by metabolic stress triggered by factors such as amino acid scarcity. Activating transcription factor-4 (ATF4) and its upstream regulator GCN2 play crucial roles in the metabolic reprogramming and functionality of CD4^+^ and CD8^+^ T cells. ATF4 can be activated by various cellular stress signals, including amino acid deprivation. While ATF4 activation may be associated with T-cell dysfunction, its role in stress adaptation presents an opportunity for therapeutic intervention—particularly in the tumor microenvironment, where T-cell exhaustion is a major challenge. In this study, we developed a strategy to harness the GCN2‒ATF4 axis in CAR-T cells. We employed an amino acid-dependent inducible promoter, which triggers ATF4-dependent gene expression to regulate CAR expression in T cells under conditions of amino acid scarcity within the tumor microenvironment. In vitro and murine xenograft models demonstrate the potential of this system to effectively restrict CAR expression to the tumor site. This targeted strategy not only enhances safety by minimizing off-tumor activity but also CAR-T-cell fitness by reducing exhaustion. By validating this pathophysiologically regulatable CAR expression system for solid tumors, our findings address key limitations of current CAR-T-cell therapies and pave the way for innovative strategies targeting solid malignancies.

## Introduction

CAR-T-cell therapies have demonstrated remarkable antitumor activity, particularly in the context of hematological malignancies [1–3], but remain largely ineffective in solid tumors [4–6]. This ineffectiveness is attributed primarily to on-target, off-tumor toxicity and to the reduced effector function of CAR-T cells caused by the complex and variable tumor microenvironment [7–9].

T-cell function is highly governed by metabolic reprogramming in this microenvironment [10], where amino acid deprivation and hypoxia are common features [11–13]. A key player in this adaptation is the activating transcription factor-4 (ATF4) pathway. It is activated under various stress conditions, including endoplasmic reticulum (ER) stress, oxidative stress, and nutrient or oxygen shortages, through the phosphorylation of PERK or GCN2, which in turn phosphorylates eIF2α, leading to increased ATF4 translation [14–17]. Once upregulated, ATF4 orchestrates transcription programs that enable cells to cope with stress by directly binding to amino acid response elements (AAREs) in target gene promoters [18]. The first evidence of ATFR4 binding to AARE was reported in 1999 [19], providing initial evidence of its role as a stress-responsive transcription factor. Since then, its critical function in regulating genes involved in metabolic adaptation and cellular stress responses has been established [20].

In addition to its role in metabolic adaptation, ATF4 plays a dual role within the tumor microenvironment, influencing both pro- and antitumor immune responses. Its upregulation is crucial for the metabolic reprogramming and functionality of CD4^+^ and CD8^+^ T cells [21–23]. Under low-arginine conditions, ATF4 confers immunosuppressive properties to activated CD4^+^ T cells, whereas its downstream effector CHOP, which is activated via the PERK‒ATF4 pathway, acts as a negative regulator of the antitumour response in CD8^+^ T cells [22, 24]. Notably, increased CHOP expression in tumor-infiltrating CD8^+^ T cells is correlated with poor clinical outcomes in advanced ovarian cancer patients [22, 24]. Additionally, ATF4 contributes to CD8^+^ T-cell exhaustion by transactivating multiple immune checkpoint genes in a PERK-JAK1-STAT3 signaling-dependent manner, further promoting immune dysfunction within tumors [25]. However, ATF4 and its upstream regulator GNC2 are also crucial for CD8^+^ T-cell survival and antitumour activity within the tumor microenvironment, highlighting its paradoxical role in immune regulation [26, 27]. Furthermore, exposure to key metabolic constraints of the tumor microenvironment, such as limited asparagine levels, further upregulates ATF4 expression in T cells, reinforcing its role as a stress sensor in immune adaptation [23].

Although ATF4 plays negative and positive roles within the tumor microenvironment, we investigated the effect of its upregulation in T cells in the context of amino acid scarcity. We optimized an amino acid response element-based promoter [28], referred to as 2xAARE-YB, to regulate CAR expression. By restricting CAR expression in T cells to the tumor microenvironment in an ATF4-dependent manner, the 2xAARE-YB system reduces the risk of on-target, off-tumor toxicity. Furthermore, while constitutive overexpression of transcription factors, such as c-Jun, enhances the potency of CAR-T cells [29, 30], their potential oncogenicity may raise concerns. Interestingly, CAR expression, along with coexpression of CAR and the c-Jun transcription factor under the control of the 2xAARE-YB promoter, not only mitigated T-cell exhaustion but also improved T-cell fitness and antitumor activity. Additionally, this ATF4-regulatable system enables pharmacological control of CAR expression, offering an alternative approach for intermittent transgene regulation. Collectively, our findings pave the way for translational interventions aimed at developing more effective and safer CAR-T-cell-based therapies against solid tumors.

## Results

### Amino acid scarcity induces the ATF4 pathway in T cells

**Fig. 1:**
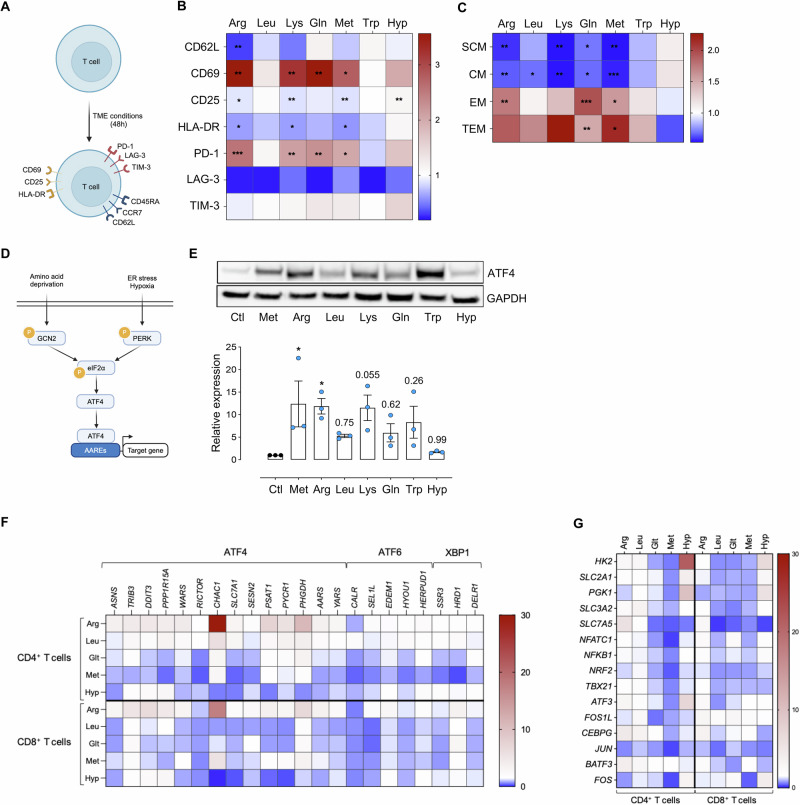
Amino acid scarcity induces ATF4 in T cells. **A** Schematic representation of the experimental model used to evaluate the T-cell immunophenotype and subsets under amino acid (AA) and oxygen restriction. **B** Heatmap illustrating the fold change in the expression of major functional T-cell markers under AA and oxygen restriction conditions compared with the control conditions. **C** Heatmap illustrating the fold changes in the proportions of stem central memory (SCM), central memory (CM), effector memory (EM), and terminal effector memory (TEM) T-cell subsets under AA and oxygen restriction conditions compared with those under control conditions. **D** Schematic representation of the ISR pathways that induce ATF4. **E** Immunoblot showing ATF4 expression in T cells after 24 h of culture under AA restriction and severe hypoxia (0.1% O_2_). The histograms in the lower panel show the relative expression under different conditions normalized to the expression of the housekeeping control GAPDH. Heatmaps illustrating the mRNA expression of genes associated with the ATF4, ATF6 and XBP1 pathways (**F**) and other T-cell-associated genes (**G**) in CD4^+^ and CD8^+^ T cells after 24 h of culture under AA restriction and severe hypoxia (0.1% O_2_). mRNA expression is represented as the fold change in expression normalized to that in the control medium. All the results are presented as the means ± SEMs of three independent healthy donors. Statistical analyses were performed with one-way ANOVA. Asterisks represent significant differences between each group and the control group (*p < 0.05, **p < 0.01, ***p < 0.001)

To harness the ATF4 pathway for enhancing CAR-T-cell functions in the solid tumor microenvironment, we utilized an in vitro model (Fig. 1A) to examine the behavior of activated CD3^+^ T cells under amino acid (AA) deprivation and severe hypoxia (0.1% _2_). Using multiparametric flow cytometry (Supplementary Fig. 1), we found that exposure of activated CD3^+^ T cells to deficiencies in arginine (Arg), leucine (Leu), lysine (Lys), methionine (Met), glutamine (Gln) and tryptophan (Trp), conditions commonly observed in the tumor microenvironment [31, 32], or severe hypoxia altered the expression of several cell surface markers associated with T-cell differentiation, activation, and exhaustion (Fig. 1B). AA scarcity led to decreased expression of L-selectin CD62L, a marker linked to stemness and the naive phenotype, which is typically downregulated within the tumor microenvironment [33], as well as reduced levels of the activation markers CD25 and HLA-DR. In contrast, the activation marker CD69, which is frequently expressed on intratumor T cells [34, 35], was significantly upregulated. The levels of the exhaustion markers PD-1 and TIM-3, but not LAG-3, were also elevated (Fig. 1B). Notably, these alterations were less pronounced in the presence of Leu and Trp scarcity. In parallel, AA deficiency and severe hypoxia also reshaped the functional phenotype of CD3^+^ T cells, particularly affecting memory and effector subsets (Fig. 1C). Both CD45RA^−^CCR7^−^ effector memory (EM) and CD45RA^+^CCR7^−^ terminal effector memory (TEM) T cells, which dominate the tumor microenvironment [36], were increased, whereas CD45RA^+^CCR7^+^ stem cell-like memory (SCM) and CD45RA^−^CCR7^+^ central memory (CM) T cells were reduced (Fig. 1C). Hence, exposure of activated CD3^+^ T cells to restricted AA and oxygen conditions shifts their immune and functional phenotype toward that of tumor-infiltrating T cells.

Next, we examined the regulation of the ATF4 pathway (Fig. 1D) in activated CD3^+^ T cells via the same in vitro model. AA deficiency was more effective than hypoxia in increasing ATF4 levels, although the extent of upregulation varied (Fig. 1E). Both AA restriction and hypoxia considerably upregulated the ATF4 target genes *TRIB3, DDIT3* and *PPP1R15A*, whereas only AA deprivation upregulated *CHAC1, PSAT1, PYCR1* and *PHGDH* (Fig. 1F). Nonetheless, Met deficiency was less efficient at inducing these genes (Fig. 1F), despite its ability to upregulate ATF4 protein levels (Fig. 1E). Additionally, target genes of other endoplasmic reticulum (ER) stress-related pathways, such as ATF6 and XBP1, were slightly upregulated, primarily in CD4^+^ T cells (Fig. 1F). Furthermore, both CD4^+^ T cells and CD8^+^ T cells presented increased expression of amino acid transport-related genes (*SLC2A1, SLC3A2* and *SLC7A5*) as well as ATF4 partners (*ATF3* and *CEPBG*), whereas activation-related genes (*NFATC1, NFKB1, NRF2, TBX21, JUN* and *FOS*) were downregulated (Fig. 1G).

Together, these results indicate that our in vitro model successfully recapitulates key features of the solid tumor microenvironment. These findings support the potential of leveraging the ATF4 pathway to improve the safety and efficacy of CAR-T-cell therapies for solid tumors.

### The ATF4 pathway is activated in melanoma-infiltrating T cells

**Fig. 2:**
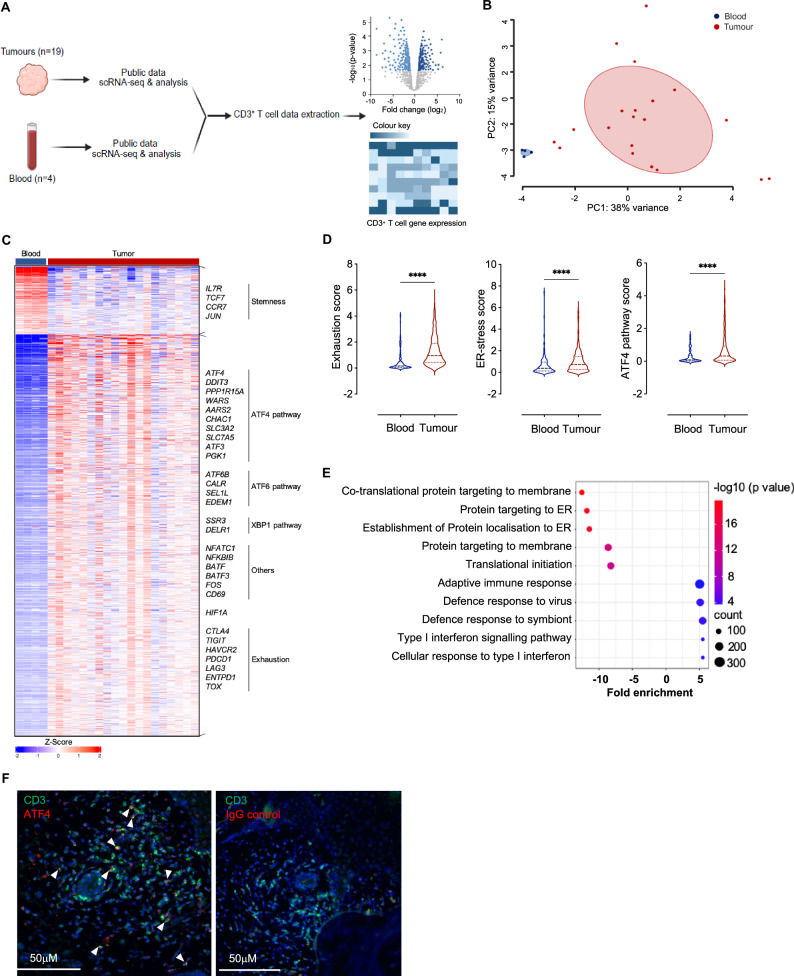
The ATF4 pathway in melanoma-infiltrating T cells. **A** Schematic representation of the public scRNA-seq data analysis workflow. **B** Principal component analysis (PCA) based on RNA expression from melanoma tumor-infiltrating T cells (TILs) and healthy donor T cells. **C** Heatmap illustrating genes differentially expressed between melanoma TILs and healthy donor T cells. **D** Violin plots of exhaustion, endoplasmic reticulum (ER) stress and ATF4 signature scores on the basis of RNA expression in melanoma TILs compared with healthy donor T cells. **E** Bubble plot illustrating pathway analysis incorporating genes downregulated and upregulated in melanoma TILs via the GO process. All results are representative of healthy donor blood (*n* = 4) and melanoma TIL (*n* = 19) samples and are presented as the mean values ± SEM. Statistical analyses were performed with two-sided Student’s t tests. Asterisks represent significant differences between TILs and blood T cells (****p < 0.0001). **F** Representative immunofluorescence images of three human primary melanomas. DAPI (nuclei, blue) and an anti-CD3 antibody (green) and either an anti-ATF4 antibody (red) or its IgG isotype control (red) are shown

To further validate our approach, we investigated the activation of the ATF4 pathway in human melanoma tumor-infiltrating lymphocytes (TILs). We leveraged publicly available single-cell RNA sequencing (scRNA-seq) data from nineteen human melanoma samples and compared them to the scRNA-seq data of blood samples from four healthy individuals. After normalizing the datasets, we extracted and analyzed gene expression data specific to CD3^+^ T cells (Fig. 2A). Despite heterogeneity among samples, principal component analysis (PCA) revealed a distinct gene signature that differentiated melanoma TILs from healthy blood-derived T cells (Fig. 2B). Differential expression analysis revealed 2609 genes whose expression was either upregulated or downregulated in melanoma TILs (Supplementary Fig. 2A).

Consistent with our in vitro findings, genes associated with the ATF4 (e.g., *ATF4, DDIT3, PPP1R15A* and *CHAC1*), ATF6 (e.g., *ATF6B, CALR, SEL1L*, and *EDEM1*) and XBP1 (e.g., *SSR3* and *DELR1*) pathways were significantly enriched in melanoma TILs compared with healthy blood T cells (Fig. 2C and Supplementary Fig. 2B). Additionally, genes involved in T-cell fate regulation (e.g., *CD69, CTLA4, TIGIT, HAVCR2* and *PDCD1*) were significantly upregulated, whereas genes associated with T-cell stemness (*IL7R, CCR7 and TCF7*) were significantly downregulated (Fig. 2C and Supplementary Fig. 2C). Pathway score analysis further revealed that, compared with healthy blood T cells, melanoma TILs presented higher exhaustion, ER stress and ATF4 signature scores (Fig. 2D). Gene Ontology (GO) analysis of biological processes revealed that pathways related to ER stress, including increased protein targeting to the membrane and ER and translation initiation, were downregulated (Fig. 2E). To corroborate the findings from the data mining, we assessed the protein expression of ATF4 in CD3^+^ T cells infiltrating primary melanoma patient biopsies. Immunofluorescence imaging confirmed that CD3^+^ T cells within the tumors expressed the ATF4 protein (Fig. 2F).

Thus, these results show that the ATF4 pathway is activated in T cells infiltrating melanomas, further supporting the potential of leveraging this pathway to enhance CAR-T-cell therapies for solid tumors.

### The 2xAARE-YB-inducible system efficiently controls CAR expression

**Fig. 3:**
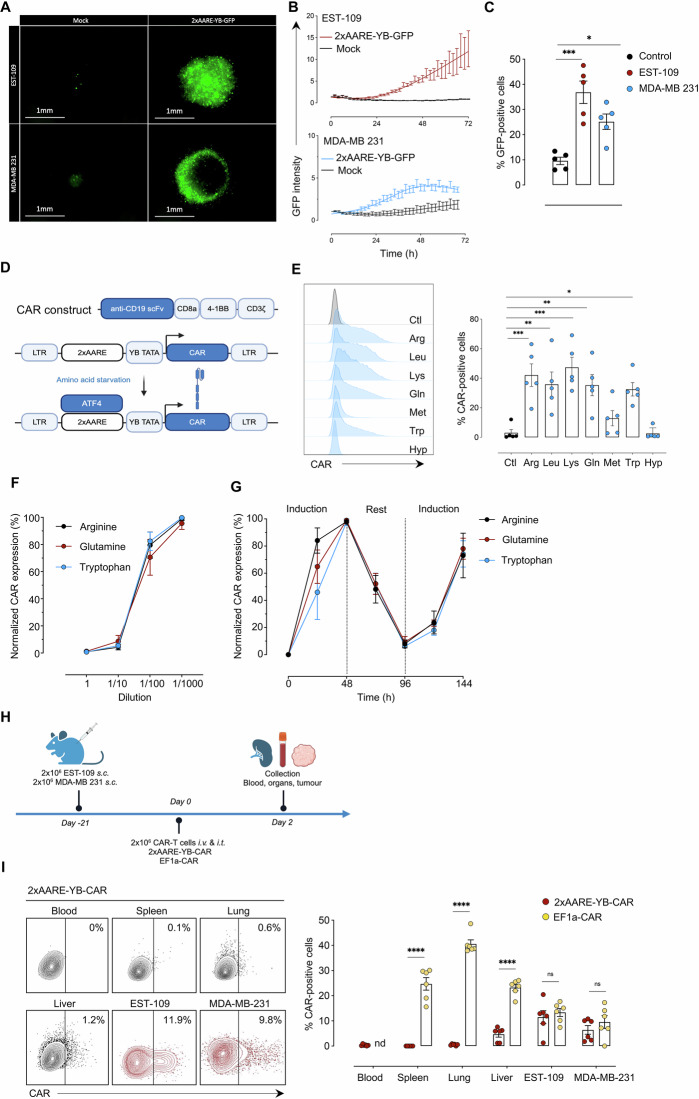
The 2xAARE-YB system induces CAR expression. **A** Representative immunofluorescence images of melanoma (EST-109) and breast cancer (MDA-MB 231) spheroids after 72 h of coculture with 2xAARE-YB-GFP-T cells in control medium. **B** GFP intensity of untransduced (mock) or 2xAARE-YB-GFP-T cells infiltrating EST-109 and MDA-MB 231 spheroids over time. **C** Bar plot illustrating the percentage of (%) GFP^+^2xAARE-YB-GFP-T cells cultured alone (Ctl) or with spheroids. **D** Schematic representation of the 2xAARE-YB-regulated CAR expression construct. **E** Representative histogram and bar plot showing the expression of CAR in 2xAARE-YB-CAR-T cells cultured under different AA restrictions. **F** Relative surface CAR expression in 2xAARE-YB-CAR-T cells cultured with increasing dilutions of AA. **G** Surface CAR expression in 2xAARE-YB-CAR-T cells at the indicated times under conditions of AA starvation, rest and re-exposure to AA-restricted conditions. In both **F** and **G**, the maximum CAR expression (%) at a 1/1000 dilution for each AA is normalized at 100%. The results are presented as the mean values ± SEM obtained from five independent healthy donors. **H** Schematic representation of the mouse experimental protocol. Groups (*n* = 2) of NXG mice (*n* = 6/group) were s.c. injected with 2×106 CD19 + EST-109 or CD19 + MDA-MB 231 cells. Tumors were developed over 21 days, after which the mice received i.v. and i.t. injections of 2xAARE-YB-CAR or EF1a-CAR-T cells. Blood, spleen, lungs, liver and tumors were collected two days later. **I** Representative contour plots and bar plots showing the percentages of (%) CAR-positive 2xAARE-YB-CAR- and EF1a-CAR-T cells in the blood, spleen, lungs, liver, CD19 + EST-109 and CD19 + MDA-MB 231 tumors. Not detected (nd). All the results are presented as the means ± SEMs of data obtained from 6 mice/group. Statistical analyses were performed with one- and two-way ANOVA. Asterisks represent significant differences between groups (*p < 0.05, **p < 0.01, ***p < 0.001, ****p < 0.0001)

The limited bioavailability of AA distinguishes the tumor microenvironment from healthy tissues and presents a promising avenue for developing CAR-T cells specific to solid tumors. Previous murine studies demonstrated that an AA-dependent inducible system known as 2xAARE triggers ATF4-dependent gene expression [28]. This system utilizes an inducible promoter composed of six copies of the *TRIB3* amino acid responsive element (AARE), which is linked to the human thymidine kinase (*TK1*) minimal promoter [28]. Since AA restriction upregulates ATF4 in activated T cells (Fig. 1E) and *TRIB3* overexpression is consistently observed across various cancers (Supplementary Fig. 3A), we employed the 2xAARE system from the *TRIB3* gene to create an ATF4-based strategy that restricts CAR expression to the TME. We engineered this system by combining AARE with the YB TATA minimal promoter, instead of *TK1*, and cloned it into a lentiviral vector (Supplementary Fig. 3B). This modified promoter, termed 2xAARE-YB, contains six AARE repeats derived from the *TRIB3* promoter and was validated for its ability to induce ATF4-dependent GFP expression in T cells infiltrating 3D spheroid models that mimic the tumor microenvironment, which is characterized by AA deprivation and hypoxia [37–39]. Coculturing EST-109 melanoma spheroids with 2xAARE-YB-GFP-expressing CD3^+^ T cells resulted in a time-dependent increase in GFP expression specifically within spheroid-infiltrating T cells (Fig. 3A–C). Similar results were obtained with MDA-MB-231 breast cancer spheroids (Fig. 3A–C).

Next, we constructed a second-generation anti-CD19-41BBz CAR under the control of the 2xAARE-YB promoter in T cells to investigate its regulation under AA-limited conditions in different tumor models harboring the same target antigen (Fig. 3D). CAR expression was induced by deficiencies in Arg, Leu, Lys, Gln, or Trp, with Met having a lesser effect, while hypoxia had no impact (Fig. 3E). Importantly, there was no unintended expression under normal conditions. Similar CAR expression levels were observed in both CD4^+^ and CD8^+^ T cells (Supplementary Fig. 3C). Given that Arg, Gln, and Trp deficiencies are frequently encountered within the tumor microenvironment [31], we further examined their impact on 2xAARE-YB-dependent CAR expression. Our results demonstrated that CAR expression increased as the concentration of these AAs decreased (Fig. 3F), with reversible expression acting as a switch that could be toggled on or off on the basis of the AA concentration (Fig. 3G). Thus, the 2xAARE-YB promoter regulates CAR expression under conditions of AA scarcity through the widely established ability of ATF4 to bind to AARE motifs.

On the basis of these findings, we evaluated the potential of the 2xAARE-YB promoter as an on-switch strategy using the ATF4-inducing drugs artesunate and L-asparaginase. These drugs induce ATF4 through endoplasmic reticulum (ER) stress and the integrated stress response (ISR), respectively, and have demonstrated antitumour properties [23, 40–42]. Artesunate and L-asparaginase successfully induced 2xAARE-YB-dependent CAR expression in T cells, with no unintended CAR expression when the cells were exposed to vehicle (Supplementary Fig. 3D). CAR expression was slightly more pronounced in CD4^+^ T cells than in CD8^+^ T cells (Supplementary Fig. 3E). These findings suggest that the 2xAARE-YB promoter has strong potential as an on-switch strategy since both drugs are able to drive significant CAR expression in T cells.

We then investigated the ability of the 2xAARE-YB system to restrict CAR expression to the tumor microenvironment in vivo. Immunodeficient NXG mice were subcutaneously (s.c.) implanted with CD19^+^ EST-109 or CD19^+^ MDA-MB 231 tumor cells. To ensure appropriate homing within the tumors, the mice were then intravenously (i.v.) and intratumorally (i.t.) injected with T cells expressing either the regulatable 2xAARE-YB-anti-CD19-CAR or the constitutive EF1a-anti-CD19-CAR. T cells were isolated from healthy tissues (lung, spleen, and liver), blood, and tumors and then analyzed for CAR expression via flow cytometry (Fig. 3H and Supplementary Fig. 4A). In contrast to EF1a-CAR-T cells, 2xAARE-YB-CAR-T cells presented almost no detectable CAR expression outside the tumors (Fig. 3I and Supplementary Fig. 4B). A slight increase in CAR expression was observed in 2xAARE-YB-CAR-T cells infiltrating the liver; this increase was primarily restricted to CD4^+^ T cells (Supplementary Fig. 4C). Importantly, in both CD19^+^-EST-109 and CD19^+^-MDA-MB 231 tumors, the 2xAARE-YB system efficiently induced CAR expression in tumor-infiltrating T cells, achieving levels comparable to those of EF1a-CAR-T cells (Fig. 3I).

These findings highlight the potential of the 2xAARE-YB system to confine CAR expression predominantly to tumor sites, making it a promising strategy for the development of safer CAR-T-cell therapies targeting solid tumors.

### Controlling CAR expression enhances T-cell fitness

**Fig. 4:**
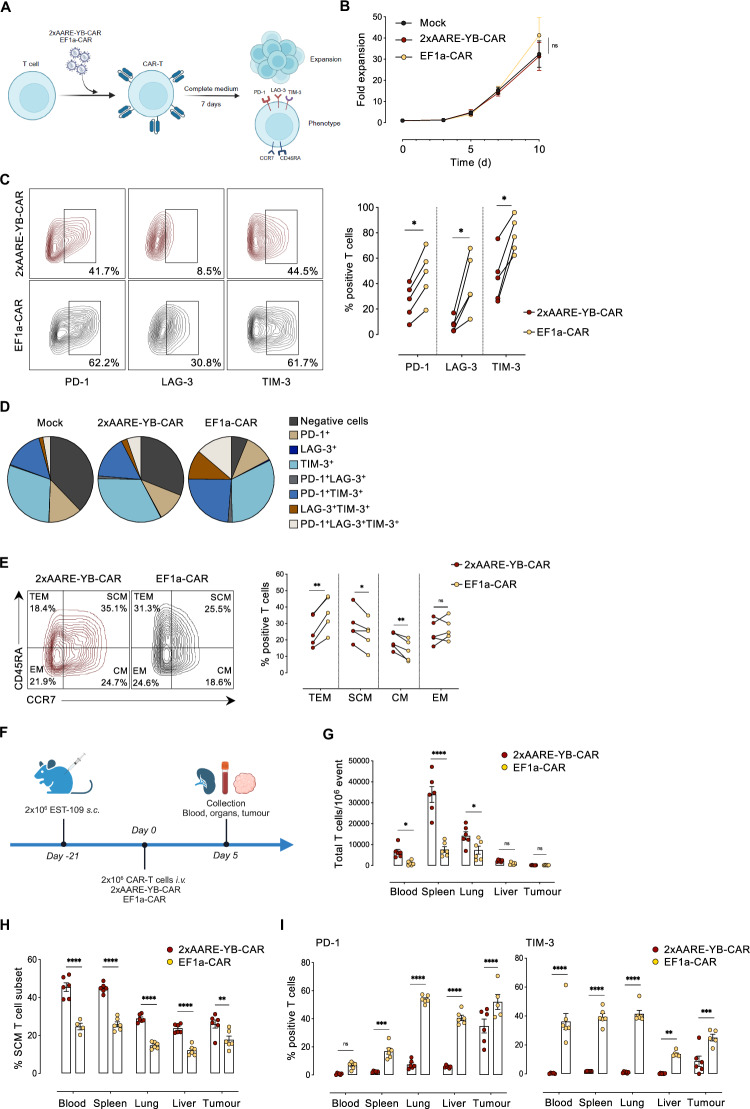
Expanded 2xAARE-YB-CAR-T cells exhibit a memory phenotype and reduced exhaustion. **A** Experimental procedure and readout of expanded CAR-T cells. **B** Fold expansion of untransduced (mock), 2xAARE-YB-CAR, or EF1A-CAR-T cells. **C** Representative contour plots and connected dot plots representing the percentages of (%) PD-1-, LAG-3- and TIM-3-positive 2xAARE-YB- and EF1a-CAR-T cells after their expansion. **D** Pie chart illustrating the percentage of (%) PD-1-, LAG-3- and TIM-3-positive 2xAARE-YB- and EF1a- CAR-T cells after their expansion as well as the percentage of (%) double- or triple-positive cells as indicated. **E** Representative contour plots and connected dot plots representing the percentages of (%) SCM, CM, EM, and TEM 2xAARE-YB-CAR or EF1a-CAR-T-cell subsets after expansion. The results are presented as the mean values ± SEM obtained from five independent healthy donors. **F** Schematic representation of the mouse experimental protocol. Groups (*n* = 2) of NXG mice (*n* = 6/group) were s.c. injected with 2×106 CD19 + EST-109 cells. Tumors were developed over 21 days, after which the mice received i.v. injections of 2xAARE-YB-CAR or EF1a-CAR-T cells. Blood, spleen, lungs, liver and tumors were collected 5 days later. **G** Bar plot illustrating the number of 2xAARE-YB-CAR and EF1a-CAR-T cells/10^6^ cells that were isolated from the blood, spleen, lungs, liver and tumors. **H** Bar plot illustrating the percentage (%) of the 2xAARE-YB-CAR and EF1a-CAR-T-cell SCM subsets. **I** Bar plot illustrating the percentage of (%) PD-1- and TIM-3-positive 2xAARE-YB-CAR-T or EF1a-CAR-T cells in the blood, spleen, lungs, liver, and tumor. All the results are presented as the means ± SEMs of data obtained from 6 mice/group. Statistical analyses were performed with one- and two-way ANOVA. Asterisks represent significant differences between groups (*p < 0.05, **p < 0.01, ***p < 0.001, ****p < 0.0001)

During CAR-T-cell manufacturing, continuous CAR expression and tonic signaling can lead to T-cell exhaustion, which often reduces T-cell fitness and potency upon administration [43–45]. Limiting CAR expression during manufacturing could help mitigate exhaustion and enhance overall T-cell fitness. To investigate this, we subjected T cells expressing either 2xAARE-YB-CAR or EF1a-CAR to a 7-day expansion protocol and then determined their expansion potential and functional phenotype (Fig. 4A). Both 2xAARE-YB-CAR-T cells and EF1a-CAR-T cells exhibited similar expansion curves (Fig. 4B). However, unlike EF1a-CAR-T cells, 2xAARE-YB-CAR-T cells presented significantly lower levels of the exhaustion markers PD-1, LAG-3 and TIM-3 (Fig. 4C). Furthermore, the proportion of 2xAARE-YB-CAR-T cells that were double- or triple-positive for PD-1, -LAG-3 or -TIM-3 was markedly decreased, resembling the levels observed in mock-treated T cells (Fig. 4D).

Stem cell-like memory T cells are currently highly valued in therapy for their self-renewal capacity, ability to generate other T-cell subsets, and enhanced engraftment potential [44, 45]. We examined whether regulating CAR expression with 2xAARE-YB would favor the development of this desirable T-cell subset. Compared with EF1a-CAR-T cells, 2xAARE-YB-CAR-T cells presented a greater proportion of SCM T cells and CM T cells, indicating a stem cell-like memory phenotype (Fig. 4E). This phenotype was observed in both CD4^+^ and CD8^+^ cells, with a stable CD4/CD8 ratio in both the 2xAARE-YB-CAR-T and EF1a-CAR-T groups (Supplementary Fig. 5A–C).

Next, we assessed whether restraining CAR expression during the expansion phase would enhance T-cell fitness in vivo. 2xAARE-YB-CAR or EF1a-CAR-T cells were injected i.v. into NXG mice bearing CD19^+^ EST-109 tumors, after which the absolute number and phenotype of T cells isolated from healthy organs, blood and tumors were analyzed (Fig. 4F). A greater number of 2xAARE-YB-CAR-T cells than EF1a-CAR-T cells were detected in the blood, spleen and lungs (Fig. 4G). Additionally, 2xAARE-YB-CAR-T cells accounted for a greater proportion of SCM T cells, confirming a more pronounced stem cell-like memory phenotype (Fig. 4H). Importantly, these cells also displayed a less exhausted profile, with significantly reduced expression of PD-1 and TIM-3 markers (Fig. 4I).

Together, these findings demonstrate that 2xAARE-YB-mediated regulation of CAR expression not only enhances the safety profile by restricting CAR expression to the solid tumor microenvironment but also improves the fitness and potency of CAR-T cells. This strategy represents a valuable approach for enhancing the therapeutic efficacy of CAR-T cells.

### Coregulation of c-Jun and CAR expression in T cells enhances potency

**Fig. 5:**
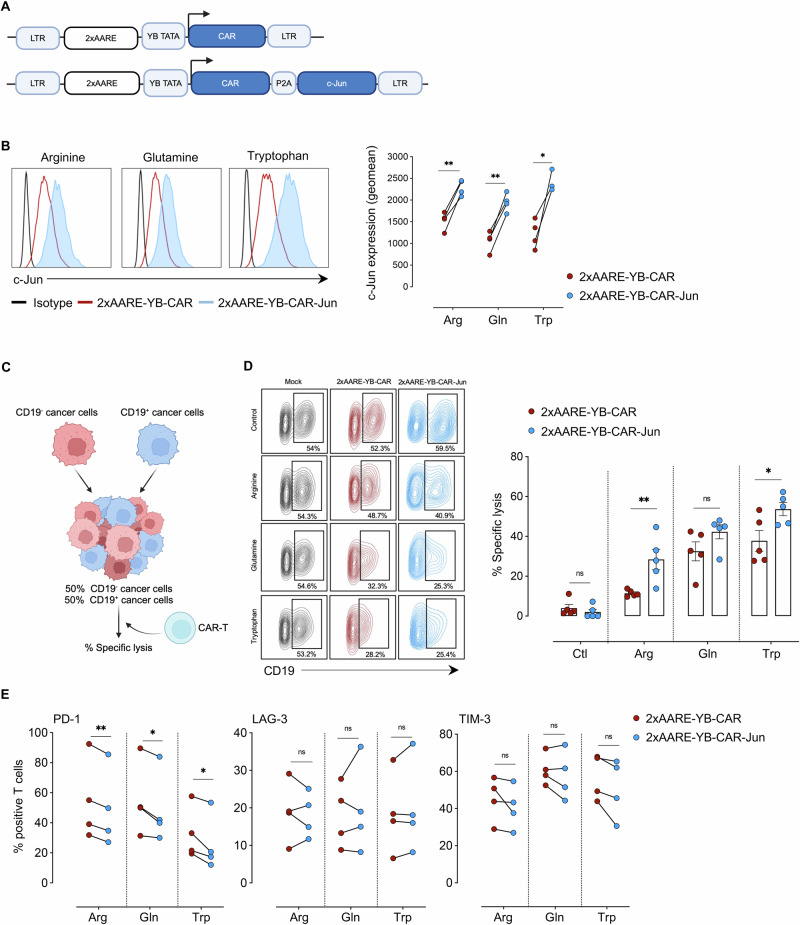
Conditional expression of c-Jun enhances the cytotoxicity of 2xAARE-YB-CAR-T cells. **A** Schematic representation of the 2xAARE-YB-CAR and 2xAARE-YB-CAR-Jun constructs. **B** Representative histograms and connected dot plots illustrating c-Jun expression in 2xAARE-YB-CAR (red) and 2xAARE-YB-CAR-Jun (blue) T cells after 48 h of amino acid restriction. **C** Schematic representation of the 2D cytotoxicity assay. **D** Representative contour plots and bar plot representing the percentage (%) of specific lysed CD19^+^ EST-109 tumor cells after 30 h of coculture with 2xAARE-YB-CAR and 2xAARE-YB-CAR-Jun T cells at an E:T ratio of 2:1 under AA-restricted conditions. **E** Connected dot plots illustrating the percentage of (%) PD-1-, LAG-3- and TIM-3-positive 2xAARE-YB-CAR or 2xAARE-YB-CAR-Jun T cells after 48 h of AA restriction. All results are presented as the mean values ± SEM obtained from four independent healthy donors. Statistical analyses were performed with one- and two-way ANOVA. Asterisks represent significant differences between groups (*p < 0.05, **p < 0.01)

Various transcription factors are known to increase the potency of CAR-T cells [29, 30]. Given that c-Jun expression is downregulated under low-AA conditions (Fig. 1G), we investigated whether the 2xAARE-YB promoter could be used to coregulate the expression of CAR and the c-Jun transcription factor. To test this hypothesis, we designed a lentiviral vector carrying a monocystronic cassette in which the 2xAARE-YB promoter controls the expression of both the anti-CD19-41BBz CAR and c-Jun sequences linked by a self-cleaving P2A peptide (Fig. 5A). Like CAR, the 2xAARE-YB promoter efficiently upregulated c-Jun expression under conditions of Arg, Gln, and Trp restriction (Fig. 5B). With comparable transduction efficiency (Supplementary Fig. 6A), both 2xAARE-YB-CAR and 2xAARE-YB-CAR-Jun exhibited similar levels of CAR expression in T cells (Supplementary Fig. 6B). Like CAR regulation, c-Jun overexpression was also reversible, with expression levels controlled by the availability of AAs (Supplementary Fig. 6C).

To evaluate the functional impact of this coregulated CAR expression, we cocultured EST-109 and CD19 + EST-109 melanoma cells at a 1:1 ratio with either mock, 2xAARE-YB-CAR-T or 2xAARE-YB-CAR-Jun-T cells under AA-restricted conditions (Supplementary Fig. 6D). We then assessed the specific lysis of CD19^+^ EST-109 cells by CAR-T cells (Fig. 5C). CD19^+^ EST-109 melanoma cells were efficiently and specifically killed by 2xAARE-YB-CAR-T cells under Gln and Trp restriction and, to a lesser extent, under Arg restriction. Notably, 2xAARE-YB-CAR-Jun-T cells were more efficient at killing CD19^+^ melanoma cells than 2xAARE-YB-CAR-T cells were (Fig. 5D) and slightly decreased the expression of the exhaustion marker PD-1 without notable effects on LAG-3 and TIM-3 (Fig. 5E). These findings demonstrate that coexpressing c-Jun with CAR under the control of the 2xAARE-YB promoter modestly reduces exhaustion and significantly enhances CAR-T-cell potency.

### 2xAARE-YB-regulated expression of CAR promotes T-cell cytotoxicity and limits exhaustion in a 3D spheroid model

**Fig. 6:**
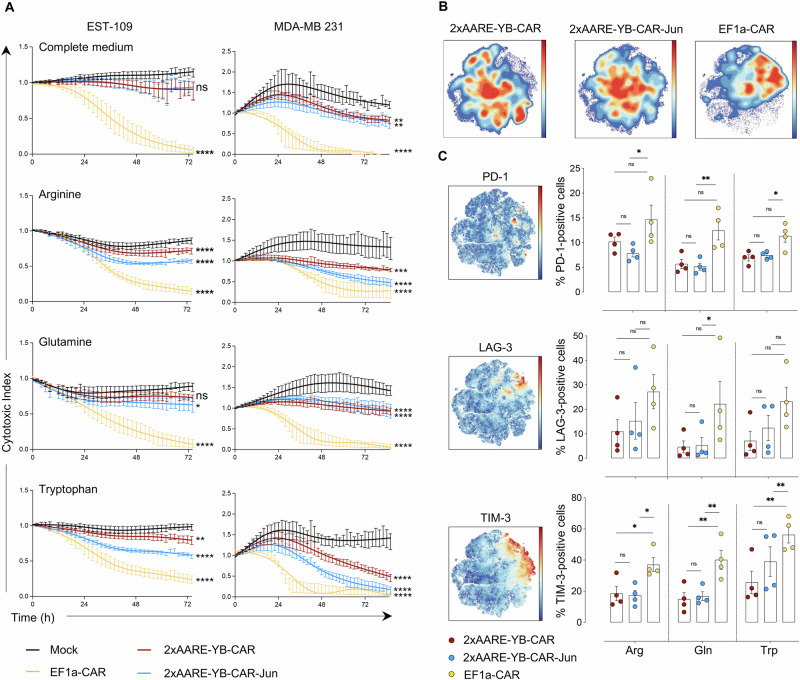
The 2xAARE-YB system generates efficient and less exhausted CAR-T cells within a 3D spheroid model. **A** Cytotoxic activity of untransduced (mock), 2xAARE-YB-CAR-Jun, 2xAARE-YB-CAR-Jun and EF1a-CAR-T cells against CD19 + EST-109 and CD19 + MDA-MB 231 spheroids over time under AA restriction. Asterisks represent significant differences between the CAR groups and mock-treated T cells (*p < 0.05, **p < 0.01, ****p < 0.0001). **B** Density plot representing the distribution of 2xAARE-YB-CAR, 2xAARE-YB-CAR-Jun and EF1a-CAR spheroid-infiltrating T cells in t-SNE after 96 h of coculture with CD19 + EST-109 spheroids and T cells under AA-restricted conditions. The color varies according to the cell abundance. **C** Scatterplots of t-SNEs and bar plots representing the intensity and percentage (%) of PD-1-, LAG-3-, and TIM-3-positive 2xAARE-YB-CAR, 2xAARE-YB-CAR-Jun and EF1a-CAR spheroid-infiltrating T cells after 96 h of coculture with CD19 + EST-109 spheroids and T cells under AA-restricted conditions. The color varies according to the surface marker abundance. All results are presented as the mean values ± SEM obtained from four independent healthy donors. Statistical analyses were performed with one- and two-way ANOVA. Asterisks represent significant differences between groups (*p < 0.05, **p < 0.01)

To evaluate the efficacy of 2xAARE-YB-regulated CAR expression in a more complex model, we used CD19 + EST-109 and CD19 + MDA-MB 231 spheroids. The spheroids were cocultured with mock, 2xAARE-YB-CAR-Jun, 2xAARE-YB-CAR-Jun, or EF1a-CAR-T cells in complete medium or in Arg-, Gln-, or Trp-deprived medium, and their growth was monitored over 72 hours. In complete medium, EF1a-CAR-T cells efficiently reduced the growth of both CD19^+^-EST-109 and -MDA-MB 231 spheroids, likely because of earlier and higher CAR expression, whereas 2xAARE-YB-CAR-T cells and 2xAARE-YB-CAR-Jun-T cells were less efficient (Fig. 6A). Under AA-deprived conditions, both 2xAARE-YB-CAR-Jun T cells and 2xAARE-YB-CAR-Jun T cells efficiently decreased the size of both spheroid models, indicating activation of the 2xAARE-YB regulatory mechanism. Notably, 2xAARE-YB-CAR-Jun-CAR-T cells exhibited significantly increased cytotoxicity (Fig. 6A). We next assessed how AA deficiency-dependent CAR expression affects the exhaustion of spheroid-infiltrating CAR-T cells. Using t-distributed stochastic neighbor embedding (tSNE), which clusters phenotypically similar cells closely together and dissimilar cells apart, we visualized the trajectories toward either the exhaustion or memory phenotype. 2xAARE-YB-CAR- and 2xAARE-YB-CAR-Jun-T cells formed clusters distinct from EF1a-CAR-T cells (Fig. 6B), with the latter displaying a more exhausted profile characterized by significant upregulation of PD-1 and TIM-3, along with a moderate increase in LAG-3 expression (Fig. 6C). 2xAARE-YB-CAR- and 2xAARE-YB-CAR-Jun-T cells also displayed a stem-like memory phenotype, marked by increased expression of CD127 and CCR7 (Supplementary Fig. 7A). This phenotype was further supported by a slight increase in the SCM T-cell subset at the expense of the TEM subset (Supplementary Fig. 7B). Additionally, the ATF4 stress-inducing drugs artesunate and L-asparaginase not only reduced the growth of CD19 + EST-109 and MDA-MB-231 spheroids (Supplementary Fig. 7C) but also enhanced the cytotoxicity of 2xAARE-YB-CAR- and 2xAARE-YB-CAR-Jun-T cells in both spheroid models (Supplementary Fig. 7D).

Together, these findings provide strong evidence that conditional CAR expression driven by the 2xAARE-YB system enables the generation of less exhausted stem cell-like memory-competent CAR-T cells. This approach also offers an alternative mechanism for time-controlled CAR expression in response to anticancer drugs.

### ATF4-dependent 2xAARE-YB-CAR-T cells efficiently control tumor growth

**Fig. 7:**
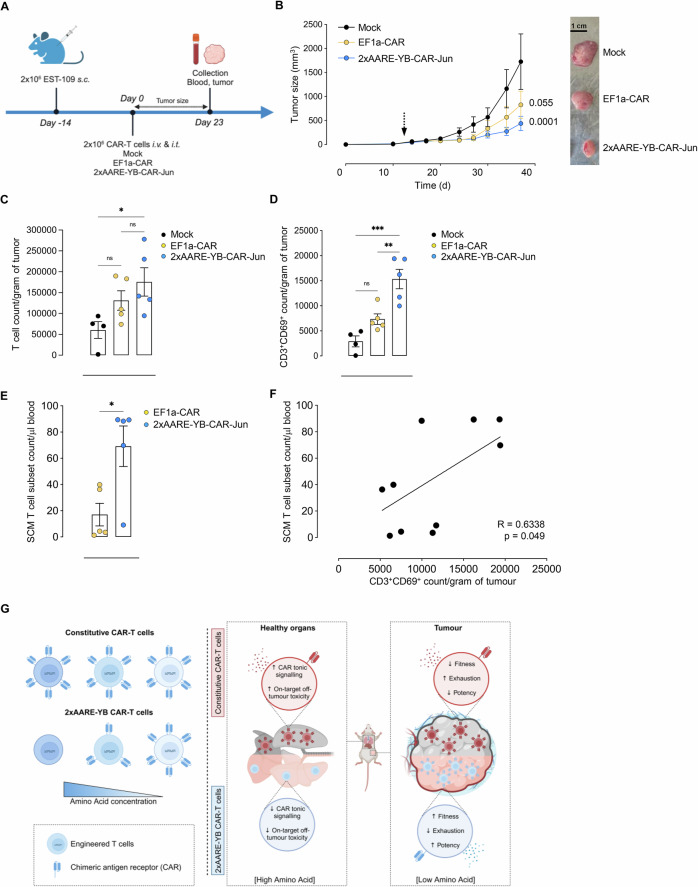
The 2xAARE-YB system generates potent antitumour CAR-T cells. **A** Schematic representation of the mouse experimental protocol. Groups (*n* = 3) of NXG mice (*n* = 5/group) were s.c. injected with 2 × 10^6^ CD19^+^ EST-109 cells. Tumors were developed over 14 days, after which the mice received i.v. and i.t. injections of mock-, 2xAARE-YB-CAR-Jun- or EF1a-CAR-T cells. Tumor size was monitored every 3–4 days. Blood and tumors were collected 23 days after injection. **B** CD19^+^ EST-109 tumor growth curves, with an arrow indicating the point at which the CAR-T cells were injected (left panel). Representative image of tumors collected on day 23 (right panel). The numeric values represent the significant differences between the CAR groups and mock-treated T cells. **C** Bar plot illustrating the absolute counts of mock-, 2xAARE-YB-CAR-Jun- and EF1a-CAR-T cells/gram of tumor cells. **D** Bar plot illustrating the absolute counts of CD69-positive mock-, 2xAARE-YB-CAR-Jun- and EF1a-CAR-T cells/gram of tumor. **E** Bar plot illustrating the absolute counts of the SCM mock-, 2xAARE-YB-CAR-Jun- and EF1a-CAR-T-cell subsets/μl of blood. **F** Scatter plot illustrating the relationship between absolute counts of the SCM T-cell subset in the blood and CD69-positive CAR-T cells in tumors. All the results are presented as the means ± SEMs of 5 mice/group. Statistical analyses were performed with two-sided Student’s t tests and one- and two-way ANOVA. Asterisks represent significant differences between groups (*p < 0.05, **p < 0.01, ***p < 0.001, ****p < 0.0001). **G** Graphical summary of the 2xAARE-YB system as an effective strategy for generating CAR-T cells targeting solid tumors

To validate the therapeutic potential of the 2xAARE-YB system against solid tumors, NXG mice bearing CD19 + EST-109 tumors were i.v. and i.t. injected with mock, 2xAARE-YB-CAR-Jun- or EF1a-CAR-T cells, and tumor size was monitored every 3–4 days for three weeks. T cells were subsequently isolated from blood and tumors, and their absolute numbers and phenotypes were determined (Fig. 7A). Compared with mock-treated T cells, 2xAARE-YB-CAR-Jun-T cells significantly reduced tumor growth, whereas EF1a-CAR-T cells had a less pronounced effect (Fig. 7B), despite similar numbers of CAR-T cells infiltrating the tumors under both conditions (Fig. 7C and Supplementary Fig. 8A). The potency of 2xAARE-YB-CAR-Jun-T cells is likely attributable to a substantial increase in the number of tumor-infiltrating activated CAR-T cells, as evidenced by elevated expression of the activation marker CD69 (Fig. 7D and Supplementary Fig. 8B). Furthermore, while the absolute counts of peripheral CD3^+^ T cells were comparable between the 2xAARE-YB-CAR-Jun- and EF1a-CAR-T-cell-treated groups (Supplementary Fig. 8C), a significant increase in the number of SCM CD3^+^ T cells was observed in the mice treated with 2xAARE-YB-CAR-Jun-T cells (Fig. 7E). Notably, this elevated SCM population in the blood correlated with the accumulation of CD69^+^ TILs in tumors (Fig. 7F). These findings suggest that the enhanced fitness of 2xAARE-YB-CAR-Jun-T cells promotes the accumulation of highly activated and cytotoxic CAR-T cells within tumors.

By validating the antitumour activity of 2xAARE-YB-CAR-Jun-T cells against solid tumors, our data support the use of the 2xAARE-YB system as a potent strategy for generating safe and efficient CAR-T cells against solid tumors (Fig. 7G).

## Discussion

Despite advancements in engineered T-cell therapies, the treatment of solid tumors continues to strive, particularly with respect to improving safety and therapeutic efficacy [46, 47]. In this study, we introduce a novel strategy that leverages the ATF4 pathway, which is activated by amino acid (AA) scarcity within the tumor microenvironment, as a transcriptional regulator of CAR expression. By spatially restricting CAR expression to this unique microenvironment, this system prevents off-tumor activation while enhancing the fitness and antitumour potency of CAR-T cells. These findings establish a pathophysiologically regulated CAR-T-cell regimen that could be widely applicable for treating solid tumors.

AA starvation activates a common cellular mechanism: the depletion of charged aminoacyl-tRNAs, which are crucial for protein synthesis. While this mechanism is shared across various amino acid deficiencies, each amino acid influences cellular processes in distinct ways, leading to unique effects on T cells that are independent of the ATF4-dependent stress response. Our results highlight these differences, as evidenced by the distinct upregulation of ATF4 and CAR expression. Specifically, methionine limitation strongly induces ATF4 expression but does not effectively upregulate its target genes, nor does it activate 2xAARE-YB-dependent CAR expression in T cells. This paradox may be attributed to factors such as promoter accessibility or the apoptotic effects of methionine starvation. Methionine, a precursor of S-adenosylmethionine (SAM), plays a crucial role in T-cell differentiation [48]. Its restriction disrupts methionine metabolism in CD8^+^ T cells, leading to increased apoptosis [49]. Therefore, the early and robust upregulation of ATF4 under methionine restriction may trigger apoptosis, halting gene transcription and limiting the expression of both ATF4 target genes and CAR.

The 2xAARE-YB system exploits the vital ATF4 pathway, which governs T-cell fate and metabolism during AA scarcity. In our experimental models, individual AA deficiencies led to proportional yet variable 2xAARE-YB-mediated ATF4-dependent CAR expression, likely due to the metabolic demands of activated T cells, which preferentially take up specific AAs to support their function [50, 51]. Previous reports highlight an essential role for ATF4 in CD8^+^ T-cell survival and tumor control [26], particularly under conditions of asparagine scarcity, which promotes CD8^+^ T-cell proliferation and fitness via ATF4-mediated metabolic rewiring [23]. However, arginine scarcity induces immunosuppressive properties in CD4^+^ T cells and limits CD8^+^ T-cell cytotoxicity [22]. In line with these findings, we observed that while arginine deficiency strongly induced 2xAARE-YB-mediated ATF4-dependent CAR expression, it did not significantly enhance CAR-T-cell cytotoxicity. Nevertheless, 2xAARE-YB-CAR-T cells retained effective antitumour activity, indicating that CAR signaling can override certain inhibitory effects of AA limitations.

Solid tumors exhibit complex metabolic conditions, often characterized by a combination of multiple AA deficiencies that vary across tumor types and patients [52, 53]. Each amino acid plays a distinct role, and its depletion—either individually or in combination—induces various metabolic changes in T cells, including CAR-T cells, which impact their function. For example, L-glutamine scarcity limits glutaminolysis and decreases glutathione levels, whereas methionine restriction lowers intracellular methionine and S-adenosylmethionine levels, both of which contribute to PD-1 expression and T-cell exhaustion [54, 55]. In contrast, L-asparagine deprivation enhances T-cell metabolism and increases antitumour activity [23]. Although the precise metabolic changes in CAR-T cells under different amino acid deficiencies are difficult to predict, we observed differential killing activity in CAR-T cells exposed to arginine, glutamine, or tryptophan deficiency. Notably, arginine deficiency was the most potent inhibitor of CAR-T-cell cytotoxic function. Additionally, we demonstrated that the 2xAARE-YB system enhances CAR-T-cell fitness under these amino acid deficiencies, likely by limiting CAR tonic signaling, as shown by Weber et al. [43]. Overall, our findings suggest that similar metabolic changes leading to exhaustion may occur in CAR-T cells within the tumor microenvironment and in the context of amino acid deficiencies. Importantly, in both melanoma and breast cancer models, 2xAARE-YB-dependent CAR expression was effectively induced, indicating that the amino acid combinations in these tumors, although undetermined, were sufficient to activate the system. Understanding the broader metabolic landscape of various solid tumors will be critical for optimizing the 2xAARE-YB system to accommodate malignancies and patient-specific characteristics.

A major challenge in CAR-T-cell therapy for solid tumors is the risk of on-target off-tumor toxicity [7], as tumor-associated antigens are often shared with normal tissues. The 2xAARE-YB system takes advantage of the unique amino acid scarcity of the tumor microenvironment to ensure the absence of CAR expression under normal physiological conditions, thereby minimizing the risk of off-tumor toxicity. Our results revealed that the use of a solid tumor microenvironment-regulatable system in which functional CAR expression is restricted to amino acid scarcity ensures the complete absence of leaky expression under physiological conditions and probably reduces the risk of on-target off-tumor toxicity. The dual hypoxia-sensing system that selectively expresses CARs under severe hypoxia in the tumor microenvironment reduces systemic on-target off-tumor cytotoxicity [56]. However, naturally hypoxic organs, such as the renal medulla and gut mucosa, can unintentionally activate CAR expression. A similar scenario might occur with the 2xAARE-YB system since certain tissues might also present low amino acid concentrations. This possibility is unlikely because a tumor-free 2xAARE-driven luciferase mouse model demonstrated that this system was not activated in any tissue under normal physiological conditions [57]. However, potential safety concerns might arise within the brief window required for complete downregulation of 2xAARE-YB-driven CAR expression. However, CD8^+^ T cells are known to home to the tumor bed and halt their migration upon encountering their target antigen [58]. Accordingly, upon encountering antigens, the likelihood of 2xAARE-YB-CAR-T cells re-entering healthy tissues during a brief window is low.

A common obstacle in CAR-T-cell therapy is constitutive CAR expression, which leads to tonic signaling that can drive T-cell exhaustion and reduce efficacy [43, 59]. In contrast, the 2xAARE-YB system, unlike constitutive expression systems, allows for a transient “rest” state during in vitro expansion. This reduces exhaustion and promotes the acquisition of a stem cell-like memory phenotype. This feature is similar to that of drug-regulatable systems, where temporary CAR downregulation enhances cell fitness and longevity [43]. The overexpression of c-Jun, a transcription factor known to enhance CAR-T-cell function, has been shown to improve fitness and efficacy in preclinical models [29], but its constitutive expression may increase safety concerns due to its oncogenic potential. In our experiments, conditional expression of c-Jun, regulated by the 2xAARE-YB system, was also effective in enhancing CAR-T-cell cytotoxicity, indicating that targeted conditional expression of oncogenic transcription factors such as c-Jun could improve CAR-T-cell potency without increasing safety risks. While c-Jun overexpression has been shown to improve CAR-T-cell antitumour activity, the underlying mechanisms remain incompletely understood. c-Jun is a critical regulator of metabolic pathways, particularly in enhancing oxidative phosphorylation, which is essential for T-cell persistence and function. It has been identified as a key regulator of the levels of mitochondrial glutaminase, an enzyme crucial for glutamine metabolism that supports the TCA cycle and oxidative phosphorylation [60]. Additionally, c-Jun negatively regulates glycolysis by inhibiting mTORC2 and glucose uptake [61, 62], as excessive glycolytic activity can lead to T-cell exhaustion [63]. Although we did not investigate the impact of conditional c-Jun expression on T-cell metabolic activity, the findings mentioned above suggest that c-Jun overexpression likely enhances 2xAARE-YB-CAR-T-cell antitumour activity through metabolic rewiring. Specifically, it may shift energy production from glycolysis to oxidative phosphorylation, thereby improving CAR-T-cell function and persistence.

Solid tumors are highly heterogeneous, with conditions evolving over time. This dynamic environment may alter AA availability and affect 2xAARE-YB-mediated CAR expression. However, the flexibility of the 2xAARE-YB system allows it to be activated not only by tumor-derived signals but also by clinically approved drugs such as artesunate and L-asparaginase. These drugs can serve as additional safeguards, providing a switch from tumor microenvironment-regulatable CAR expression to an on/off switch for ATF4-inductable CAR expression while also offering synergistic antitumor effects [41, 42]. Although drug-induced CAR expression is systemic and not confined to the tumor microenvironment, this potential risk of off-tumor toxicity could be managed by discontinuing the drug, maintaining the overall safety profile of the therapy. Thus, the 2xAARE-YB system is a “multiedged sword” strategy that is distinct from most existing inducible systems [43, 64, 65].

In this study, we aimed to provide an initial proof-of-concept for the use of the 2xAARE-YB system for CAR-T-cell regimens for solid tumors. To study the applicability of this system, we used a single CAR in xenograft tumor models with immunocompromised mice, which facilitated human tumor engraftment and human CAR-T-cell injection. We employed a second-generation CAR targeting CD19 to evaluate the fitness and antitumor capacity of 2xAARE-YB-regulated CAR-T cells. Although the CD19 antigen is not associated with solid tumors, its use provides homogenous unbiased 2xAARE-YB-regulated CAR-T cells that, in addition to mitigating experimental variations related to the use of two different tumor models, enable a clear comparison with constitutive CAR-T cells, which would have been otherwise difficult. For example, a CAR targeting the ErbB/HER family, which, in addition to tumors, is expressed in healthy tissues, would have led to rapid on-target, off-tumor toxicity in mice [64]. However, the translational relevance of our study warrants further investigation.

Employing CAR-T cells targeting clinically relevant solid tumor antigens is mandatory to validate the capacity of our system to mitigate on-target, off-tumor toxicity. The use of different models of solid tumors displaying variable levels of target antigen is needed to broaden the applicability of the 2xAARE-YB system to develop effective CAR-T-cell regimens for solid tumors. While the NXG model replicates key aspects of the solid tumor microenvironment, it lacks several tumor-associated immune cells commonly found in human tumors. The use of the 2xAARE-YB-CAR approach in other CARs and tumor models, potentially including syngeneic mouse CAR-T cells in immunocompetent preclinical or humanized mouse models of tumors, would provide the final proof-of-concept for the clinical translation of our system.

Our findings highlight the potential of the 2xAARE-YB promoter as a physiologically inducible system for regulating CAR expression, offering a strategy to mitigate CAR-T-cell exhaustion during in vitro expansion while ensuring controlled expression within solid tumors in vivo. Compared with conventional larger CAR promoters, its compact 235-Bp sequence reduces cloning complexity, facilitating large-scale clinical applications. 2xAARE recruits endogenous transcription factors without introducing heterologous or chimeric elements, thereby minimizing the risk of immune rejection. Additionally, the absence of CpG sites reduces the likelihood of immune responses triggered by methylation. However, the potential risk of methylation-induced silencing of transgene expression warrants further investigation. As with any emerging biotechnology, rigorous preclinical evaluation is needed to assess the long-term stability, safety and efficacy of the 2xAARE-YB-CAR system.

Nevertheless, our approach represents a promising advancement in addressing the challenges of CAR-T-cell therapy for solid tumors, balancing precision, safety, and therapeutic efficacy. By leveraging the metabolic landscape of tumors to regulate CAR expression, the 2xAARE-YB system lays the groundwork for next-generation, physiologically responsive CAR-T-cell therapies, potentially enabling more effective and personalized cancer treatments.

## Materials and methods

### Key resources

A detailed list of key resources for all materials and reagents used in this study is provided in Supplementary Table 1.

### Cell lines

MDA-MB 231 (HTB-26) cells were purchased from ATCC, and ESTDAB-109 melanoma cells (UKRV-Mel-2, referred to as EST-109 hereafter) were a kind gift from Federico Garrido Torres-Puchol (Granada University). The cell lines were maintained in Roswell Park Memorial Institute medium (RPMI; Gibco) supplemented with 10% fetal bovine serum (FBS; Gibco), 1X GlutaMAX (Gibco) and 1X penicillin‒streptomycin (Gibco) (cancer cell medium). The Mycoplasma strains of the cell lines were tested monthly via the MycoBlue Mycoplasma Detector (Vazyme).

### Primary human T cells

Peripheral blood mononuclear cells (PBMCs) were isolated from whole blood samples from healthy donors via Ficoll-Hypaque. The donors signed an informed consent form following the human ethics committee “Comité consultatif pour la protection des personnes dans les recherches biomédicales” - Saint Louis Hospital, Paris, France, and the study was approved by the institution. Primary human CD3^+^ T cells were subsequently isolated from PBMCs via negative selection via a Pan T-cell isolation kit (Miltenyi Biotec).

### Mice

Male NXG (^NOD‒Prkdcscid‒IL2rgTm1^) mice were purchased from Janvier Labs. All were maintained in pathogen-free conditions and were adults of 6–8 weeks of age and approximately 20–22 g in weight. The experimental protocols were approved by the Saint-Louis Research Institute Animal Care and Use Committee (committee Paris-Nord/n°121 (agreement APAFIS #43896-2023062108019955 v6)).

### CAR construct and lentiviral production

The self-inactivated (SIN) lentiviral vector plasmid (pLV), containing inducible cassettes of interest, was cloned via NEBuilder HiFi DNA Assembly (New England Biolabs) insertion of synthesized or PCR-amplified [2xAAREYB-transgene ( ± polyA)] cassettes into a destination vector containing restriction sites within the lentiviral genome. The cDNA sequences of the anti-CD19-41BBz CAR (depicted in Supplementary Table 2) and c-jun transgenes (NM_002228.4) were cloned under the control of an EF1a or 2xAARE-YB TATA gene promoter. Recombinant particles were obtained as previously described [66]. Nonreplicating lentiviral particles (LVs) were produced in HEK-293T cells (ATCC-CRL-11268) via a standard second-generation protocol [66]. Briefly, cells were cotransfected with the pLV and trans-complementation plasmids p8.91 (packaging plasmid encoding Gag-Pol, Tat, and Rev) and pVSVg (VSV-G envelope expression plasmid). Briefly, 1.2 × 10^5^ cells/cm^2^ were seeded 24 h prior to cotransfection. For each of the 10^6^ seeded cells, 1.5 µg of pLV, 1.5 µg of p8.91 and 0.75 µg of pVSVg plasmids were cotransfected via the calcium phosphate method. The media were changed 5 h later, and the LV-containing media were collected 48 h posttransfection. For ex vivo T-cell engineering, the supernatant was passed through a 0.2 µm filter and ultracentrifuged at 60,000 × g and 4 °C for 1 h 30. Pelleted particles were concentrated 1000 times in PBS and stored at -80 °C. Recombinant particles were titrated by measurement of p24 viral protein via ELISA (Kit ref. 0801008, Zeptometrix). In our experiments, we considered that 1 pg of p24 is equivalent to 100 transduction units (TUs).

### Human T-cell activation and culture

Purified CD3^+^ T cells were resuspended at a concentration of 0.5x10^6^ T cells/ml in T-cell medium (CTS^tm^ OpTmizer (Thermo Fisher Scientific)) supplemented with 1X GlutaMAX (Gibco), 1X penicillin‒streptomycin (Gibco) and 100 U/ml recombinant human IL-2 (Miltenyi Biotec). The cells were then stimulated with Human T-Activator CD3/CD28 (Thermo Fisher Scientific) at a bead:cell ratio of 2:1 for in vivo experiments or with 12.5 µl/ml ImmunoCult Human CD3/CD28/CD2 T-cell activator (STEMCELL technologies) for in vitro experiments. After 48 h, the T cells were transduced, and 72 h postactivation, the beads were removed by magnetic separation (if the T cells were activated with the human T-activator CD3/CD28). Engineered T cells were fed fresh T-cell expansion medium (CTS^tm^ OpTmizer (Thermo Fisher Scientific) supplemented with 2.5% human serum (Gibco), 1X GlutaMAX (Gibco), 1X penicillin‒streptomycin (Gibco) and 100 U/ml recombinant human IL-2 (Miltenyi Biotec)) and were maintained at a density of 0.5 x 10^6^ T cells/ml.

### Lentiviral transduction

CD3^+^ T cells were transduced with lentiviral vectors 48 h postactivation. Briefly, lentivirus concentrates were added to the medium at a multiplicity of infection (MOI) of 10. After 24 h, the cells were washed with fresh T-cell expansion medium and expanded. To obtain populations of EST-109 and MDA-MB 231 cells expressing the CD19 antigen, the cells were transduced with a “TRAP-CD19” lentiviral vector carrying the fusion gene containing splice acceptor sequences, a T2A sequence (autocatalytic peptide) encoding the CD19 cDNA sequence, a P2A sequence (autocatalytic peptide) and the bsd gene sequence (blasticidin-S deaminase). This vector allows the selection of blasticidin-resistant cells expressing the CD19 antigen under a cellular promoter, ensuring the stability of transgene expression over time. In brief, after EST-109 and MDA-MB-231 cells were transduced with this vector (MOI of 10) and selected with blasticidin, resistant cells were labeled with an anti-CD19 antibody, and cells highly expressing the CD19 antigen were sorted and amplified. The stability of CD19 expression by EST-109 and MDA-MB 231 cells was regularly checked.

### In vitro studies under amino acid scarcity and hypoxia

Following CD3^+^ T-cell activation, the cells were washed twice in PBS and resuspended in media deprived of a single amino acid (RPMI depleted of arginine, leucine, lysine, methionine, glutamine or tryptophan supplemented with 5% FBS (Gibco), 1X L-glutamine (Gibco; except for glutamine-restricted conditions), 1X penicillin‒streptomycin (Gibco), 100 U/ml recombinant human IL-2 (Miltenyi Biotec) and a 1/1000 RPMI concentration of the restricted amino acid (reagent and concentrations listed in Supplementary Table 3)) or a control medium (RPMI supplemented with 10% SVF (Gibco)), 1X L-glutamine (Gibco), 1X penicillin‒streptomycin (Gibco) and 100 U/ml recombinant human IL-2 (Miltenyi Biotec) at a concentration of 0.5x10^6^ cells/ml for 24 or 48 h. For hypoxic conditions, the cells were incubated in control medium in a hypoxia incubator chamber (STEMCELL Technologies) purged at 25 L/min for 5 min with gas containing 0.1% O_2_, 5% CO_2_ and nitrogen as a balance. For all the experiments, the cells were seeded at a concentration of 0.5x10^6^ cells/ml and incubated for 24 or 48 h. The cells were then harvested for further experiments.

### Flow cytometry

T cells were washed in FACS buffer (PBS + 0.5% FBS) and stained with fluorophore-conjugated cell-surface antibodies for 15 min at room temperature. The cells were then washed twice with FACS buffer before flow cytometry analysis. To evaluate CAR expression, the cells were incubated with CD19 CAR detection reagent (Miltenyi Biotec) at a 1/500 dilution for 15 min at room temperature and then washed twice with FACS buffer before proceeding to the abovementioned cell-surface staining in addition to the antibiotin antibody REAfinity (clone REA746, Miltenyi Biotec). Cell-surface antibodies were used at a 1/100 dilution, with the exception of the antibiotin antibody, which was used at a 1/200 dilution. Intracellular staining was performed with the same cell-surface staining protocol, after which the cells were fixed, permeabilized and stained via the BD Cytofix/Cytoperm Fixation/Permeabilization Kit (BD Biosciences) according to the manufacturer’s protocol. Intracellular antibodies were used at a 1:50 dilution, and LIVE/DEAD solution was used at a 1/500 dilution. The cells were acquired on a BD LSR Fortessa and analyzed via FlowJo v.10.9 software. The antibodies used are listed in Supplementary Table 1.

### Quantitative reverse transcription PCR

Total RNA was individually extracted from CD8^+^ and CD4^+^ T cells via the RNeasy Plus Mini Kit (Qiagen). Two micrograms of total RNA was subsequently reverse transcribed via a high-capacity cDNA reverse transcription kit (Thermo Fisher Scientific). The resulting cDNA (5 ng) was subjected to qPCR analysis with Fast SYBR Green Master Mix (Thermo Fisher Scientific) along with specific primers (see Supplementary Table 4) on a CFX384 Touch Real-Time PCR Detection System (Bio-Rad Laboratories). Each independent experiment was conducted with three technical replicates, and the expression values of each replicate were normalized against *Gusb* (beta-glucuronidase) mRNA expression via the 2-ΔΔCt method. Target mRNA expression under tumor microenvironment conditions was compared to that of the respective control (control medium) as a fold change.

### Immunoblotting

T cells were lysed with 1X RIPA buffer (Thermo Fisher Scientific). Proteins from each sample were subsequently separated on a 10% SDS‒PAGE gel under reducing conditions and transferred onto a nitrocellulose membrane (using Invitrogen™ iBlot™ 2). The membranes were then blocked for 1 h with blocking solution (1X Tris-buffered saline, 0.1% Tween (TBST; Thermo Fisher Scientific) supplemented with 2% BSA) at room temperature and then incubated with blocking solution containing the following primary antibodies: 1/1000 diluted anti-ATF4 antibody (clone D4B8, Cell Signaling Technology) or 1/1000 diluted anti-GAPDH antibody (clone 6C5, Abcam) in TBST overnight at 4 °C. The next day, the membranes were washed in TBST and incubated for 1 h with 1/3000 diluted HRP-conjugated secondary antibody (Bio-Rad) in TBST before being washed again with TBST. The membranes were then incubated for 5 min with SuperSignal West Pico PLUS Chemiluminescent Substrate reagents (Thermo Fisher Scientific) before being analyzed via the ImageQuant™ LAS 4000/4010 system (GE Healthcare).

### Immunofluorescence imaging

Formalin-fixed paraffin-embedded (FFPE) melanoma sections were prepared from biopsies from melanoma patients, deparaffinized, rehydrated, washed and then stained with two staining cycles. First, the samples were subjected to antigen retrieval (AR) with citrate buffer (pH 9), blocked with blocking buffer (ARD1001EA, Akoya), incubated with an anti-CD3 antibody (ab11089, Abcam), incubated with a secondary antibody against Akoya (ARH1001EA, Akoya), and then incubated with Opal 520 (1:250) (OP001001, Akoya). The second staining cycle included microwave treatment with citrate buffer (pH 9), blocking with blocking buffer (ARD1001EA, Akoya), incubation with an anti-ATF4 antibody (ab31390, Abcam) (1:50), incubation with the secondary antibody described above, and incubation with Opal 570 (1:250) (OP001003, Akoya). The slides were then counterstained with 1X DAPI (FP1490, Akoya) and enclosed with Vectashield Vibrance (H-1700, Eurobio Scientific). Imaging was performed with the slide scanner lamina de 3Dhistech. A whole slide scan was first acquired at 20x magnification across the full emission light spectrum in each filter tube: DAPI (450–470 nm), FITC (505–545 nm), and TRITC (600–650 nm). Images were then analyzed via ImageJ software (v1.51).

### 2D cytotoxicity assay

**A total of** 5 x 10^3^ CD19^−^ and 5 x 10^3^ CD19^+^ EST-109 melanoma cells were cocultured with 20.10^3^ CAR-T cells in 200 μl of control media or single amino acid-restricted media in flat bottom 96-well plates for 30 h. The target cells were then detached with 0.05% trypsin-EDTA (Gibco), acquired on a BD Canto II (BD Biosciences) and analyzed via FlowJo (10.9) software. Specific lysis (%) was calculated as (1-(control ratio/experimental ratio) × 100), where the control ratio corresponds to target cells cocultured with untransduced (mock) T cells.

### Spheroid formation

**A total of** 5x10^3^ EST-109 melanoma cells or MDA-MB 231 breast cancer cells were seeded in 150μl of spheroid media (RPMI supplemented with 5% FBS (Gibco), 1X GlutaMAX (Gibco), 1X penicillin‒streptomycin (Gibco) and 2.5% Matrigel basement membrane matrix (Corning)) in ultralow attachment (ULA) round-bottomed 96-well plates (PHCBI) and then spinoculated at 200 × g for 3 mins. The spheroids were subsequently grown in spheroid media for 4 days before use.

### 3D IncuCyte promoter induction assay

EST-109 or MDA-MB 231 spheroids were washed once in PBS and transferred to round-bottom 96-well plates containing 200 μl of RPMI (Gibco) supplemented with 5% FBS (Gibco), 1× GlutaMAX (Gibco), and 1× penicillin‒streptomycin (Gibco). A total of 20 x 10^3^ untransduced (mock) T cells or 2xAARE-YB-GFP T cells were then added to each spheroid. The fluorescence was monitored every 2 h with a 10x objective via an IncuCyte system (Essen Bioscience), which was housed in a cell culture incubator at 37 °C and 5% CO_2_, and a whole-well image was taken at each time point. The total integrated GFP intensity was quantified via IncuCyte software (Essen Bioscience). All the data were normalized to the first time point and plotted as the fold change in GFP fluorescence over time. After 72 h, spheroids were harvested, washed and dissociated for quantifying GFP expression by T cells in comparison to 2xAARE-YB-GFP T cells cultured for 72 h alone via flow cytometry.

### 3D IncuCyte cytotoxicity assay

In this experimental setting, cancer cells constitutively expressed GFP. EST-109 melanoma or MDA-MB 231 breast cancer spheroids were rinsed once in PBS and then transferred to round-bottomed 96-well plates containing 200 μl of control media or different single amino acid-restricted media. For the drug experiments, control media supplemented with artesunate (1 µM) or L-asparaginase (5 U/ml) were used. Subsequently, 20 x 10^3^ untransduced (mock), 2xAARE-YB-CAR, 2xAARE-YB-CAR-Jun, or EF1a-CAR-T cells were added to each spheroid. Fluorescence was assessed every 2 h via a 10x objective on the IncuCyte system (Essen Bioscience), which was located within a cell culture incubator set at 37 °C with 5% CO2, and a whole-well image was captured at each time point. The size of the largest GFP object was determined via Incucyte software (Essen Bioscience). All data were normalized to the initial time point and represented as the fold change in the largest GFP object over time. After 96 h, spheroids were collected, washed, and dissociated for analysis of the infiltrating T-cell phenotype via flow cytometry.

### Mouse studies

Tumor cell lines (2 × 10^6^ cells in PBS) were subcutaneously (s.c.) injected into male mice that were six to eight weeks of age. Once the tumors were palpable, the length (L) and width (W) of the tumors were measured every 3–4 days via a digital caliper, and the tumor volume was calculated via the following formula: (LxW^2^)*0.52. The indicated doses of mock or CAR-T cells were injected in 150 µl of PBS through the tail vein (i.v.) via a 30 G needle, and 50 µl of PBS was injected into the cells via the intratumoral (i.t.) route. At the end of each experiment, the mice were sacrificed, and samples were collected for further experiments. Briefly, blood samples were taken from the submandibular vein and transferred to 1.5 ml tubes containing 30 µl EDTA, and healthy tissues as well as tumors were kept in RPMI supplemented with 10% SVF (Gibco). For assessment of antitumour CAR-T-cell efficacy, the tumors were weighed. Then, the blood samples were incubated for 5 min with 1 ml of ACK lysis buffer (Gibco) to remove red blood cells. Tumors and healthy tissues were then minced via sharp scissors, and single-cell suspensions were obtained by incubating minced tissues for 1 h at 37 °C with 250 U/ml collagenase IV (Gibco) and 0.1 mg/ml DNase I (Gibco). The released cells were passed through a 70μm cell strainer prior to subsequent antibody staining and flow cytometry analysis of single cells from the blood, healthy tissues and tumors.

### Computational analysis

Normalized expression datasets from melanoma patients and healthy blood donors were downloaded from the Broad Institute Single Cell Portal (https://singlecell.broadinstitute.org/single_cell). Data from CD3^+^ T cells were extracted, grouped and normalized via R version 4.2.2 (https://www.r-project.org/). Principal component analysis was carried out on normalized data via the pca2d package (R version 4.2.2). Differential gene expression analyses between healthy blood CD3^+^ T cells and melanoma CD3^+^ T cells (TILs) were conducted via DESeq2 version 3.19 (FDR cutoff=0.05, minimum fold change=1.5). Gene set enrichment analysis (GSEA) was conducted to determine upregulated and downregulated cellular pathways (pathway significance cutoff: FDR = 0.05) between healthy blood CD3^+^ T cells and melanoma CD3^+^ T cells (TILs). The exhaustion, ER stress and ATF4 pathway scores were calculated on the basis of the expression of predetermined exhaustion, ER stress and ATF4 pathway signature genes, respectively (see Supplementary Table 5).

### Statistics

Statistical analyses were conducted via GraphPad Prism 10. Details regarding sample size and plot descriptions can be found in the corresponding figure legends for each analysis. To assess the statistical differences between two groups, either an unpaired Student’s *t* test or a Mann‒Whitney U test was employed. Statistical differences among three or more groups were analyzed via analysis of variance (one-way or two-way ANOVA) with the appropriate multiple comparison tests. In the plots, significance is denoted by *p* values less than 0.05.

## Supplementary information

Supplementary Materials

Unprocessed Images

## Data Availability

A source data file for all the figures and supplementary figures is provided with this paper.

## References

[1] Maude SL, Frey N, Shaw PA, Aplenc R, Barrett DM, Bunin NJ, et al. Chimeric antigen receptor T cells for sustained remissions in leukemia. N Engl J Med. 2014;371:1507–17. 25317870 10.1056/NEJMoa1407222 PMC4267531

[2] Cappell KM, Sherry RM, Yang JC, Goff SL, Vanasse DA, McIntyre L, et al. Long-term follow-up of anti-CD19 chimeric antigen receptor T-cell therapy. J Clin Oncol. 2020;38:3805–15. 33021872 10.1200/JCO.20.01467 PMC7655016

[3] Holstein SA, Lunning MA. CAR T-Cell Therapy in hematologic malignancies: a voyage in progress. Clin Pharmacol Ther. 2020;107:112–22. 31622496 10.1002/cpt.1674

[4] Zhang Y, Qin D, Shou AC, Liu Y, Wang Y, Zhou L. Exploring CAR-T-Cell therapy side effects: mechanisms and management strategies. J Clin Med. 2023;12:6124. 37834768 10.3390/jcm12196124 PMC10573998

[5] Saleki K, Mohamadi MH, Alijanizadeh P, Rezaei N. Neurological adverse effects of chimeric antigen receptor T-cell therapy. Expert Rev Clin Immunol. 2023;19:1361–83. 37578341 10.1080/1744666X.2023.2248390

[6] Kankeu Fonkoua LA, Sirpilla O, Sakemura R, Siegler EL, Kenderian SS. CAR T-cell therapy and the tumor microenvironment: Current challenges and opportunities. Mol Ther Oncol. 2022;25:69–77. 10.1016/j.omto.2022.03.009 PMC8980704 35434273

[7] Flugel CL, Majzner RG, Krenciute G, Dotti G, Riddell SR, Wagner DL, et al. Overcoming on-target, off-tumor toxicity of CAR T-cell therapy for solid tumors. Nature Rev Clin Oncol. 2023;20:49–62. 36418477 10.1038/s41571-022-00704-3 PMC10278599

[8] Sterner RC, Sterner RM. CAR-T-cell therapy: current limitations and potential strategies. Blood Cancer J. 2021;11:69. 33824268 10.1038/s41408-021-00459-7 PMC8024391

[9] Delgoffe GM, Xu C, Mackall CL, Green MR, Gottschalk S, Speiser DE, et al. The role of exhaustion in CAR T-cell therapy. Cancer Cell. 2021;39:885–8. 34256903 10.1016/j.ccell.2021.06.012

[10] Ma S, Ming Y, Wu J, Cui G. Cellular metabolism regulates the differentiation and function of T-cell subsets. Cellular Mol Immunol. 2024;21:419–35. 38565887 10.1038/s41423-024-01148-8 PMC11061161

[11] Zhang X, Song W, Gao Y, Zhang Y, Zhao Y, Hao S, et al. The role of tumor metabolic reprogramming in tumor immunity. Int J Mol Sci. 2023;24:17422. 38139250 10.3390/ijms242417422 PMC10743965

[12] Eales KL, Hollinshead KER, Tennant DA. Hypoxia and metabolic adaptation of cancer cells. Oncogenesis. 2016;5:e190. 26807645 10.1038/oncsis.2015.50 PMC4728679

[13] Wang Z, Wu X, Chen H-N, Wang K. Amino acid metabolic reprogramming in tumor metastatic colonization. Frontiers Oncol. 2023;13:1123192. 10.3389/fonc.2023.1123192 PMC10043324 36998464

[14] Ye J, Koumenis C. ATF4, an ER Stress and hypoxia-inducible transcription factor and its potential role in hypoxia tolerance and tumorigenesis. Current Mol Med. 2009;9:411–6. 10.2174/156652409788167096 19519398

[15] Rozpedek W, Pytel D, Mucha B, Leszczynska H, Diehl JA, Majsterek I. The Role of the PERK/eIF2α/ATF4/CHOP signaling pathway in tumor progression during endoplasmic reticulum stress. Current Mol Med. 2016;16:533–44. 10.2174/1566524016666160523143937 PMC5008685 27211800

[16] Kilberg MS, Shan J, Su N. ATF4-dependent transcription mediates signaling of amino acid limitation. Trends Endocrinol Metab TEM. 2009;20:436–43. 19800252 10.1016/j.tem.2009.05.008 PMC3587693

[17] Siu F, Bain PJ, LeBlanc-Chaffin R, Chen H, Kilberg MS. ATF4 Is a Mediator of the nutrient-sensing response pathway that activates the human asparagine synthetase gene. J Biol Chem. 2002;277:24120–7. 11960987 10.1074/jbc.M201959200

[18] B’chir W, Maurin A-C, Carraro V, Averous J, Jousse C, Muranishi Y, et al. The eIF2α/ATF4 pathway is essential for stress-induced autophagy gene expression. Nucleic Acids Res. 2013;41:7683–99. 23804767 10.1093/nar/gkt563 PMC3763548

[19] Fawcett TW, Martindale JL, Guyton KZ, Hai T, Holbrook NJ. Complexes containing activating transcription factor (ATF)/cAMP-responsive-element-binding protein (CREB) interact with the CCAAT/enhancer-binding protein (C/EBP)-ATF composite site to regulate Gadd153 expression during the stress response. Biochem J. 1999;339:135–41. 10085237 PMC1220137

[20] Carraro V, Maurin AC, Lambert-Langlais S, Averous J, Chaveroux C, Parry L, et al. Amino acid availability controls TRB3 transcription in liver through the GCN2/eIF2alpha/ATF4 pathway. PLoS ONE. 2010;5:e15716. 21203563 10.1371/journal.pone.0015716 PMC3006201

[21] Yang X, Xia R, Yue C, Zhai W, Du W, Yang Q, et al. ATF4 Regulates CD4+ T-Cell Immune Responses through Metabolic Reprogramming. Cell Rep. 2018;23:1754–66. 29742431 10.1016/j.celrep.2018.04.032 PMC6051420

[22] Zou Z, Cheng Q, Zhou J, Guo C, Hadjinicolaou AV, Salio M, et al. ATF4-SLC7A11-GSH axis mediates the acquisition of immunosuppressive properties by activated CD4+ T cells in low arginine condition. Cell Rep. 2024;43:113995. 38527061 10.1016/j.celrep.2024.113995

[23] Gnanaprakasam JNR, Kushwaha B, Liu L, Chen X, Kang S, Wang T, et al. Asparagine restriction enhances CD8+ T-cell metabolic fitness and antitumoral functionality through an NRF2-dependent stress response. Nature Metab. 2023;5:1423–39. 37550596 10.1038/s42255-023-00856-1 PMC10447245

[24] Cao Y, Trillo-Tinoco J, Sierra RA, Anadon C, Dai W, Mohamed E, et al. ER stress-induced mediator C/EBP homologous protein thwarts effector T-cell activity in tumors through T-bet repression. Nat Commun. 2019;10:1280. 30894532 10.1038/s41467-019-09263-1 PMC6426975

[25] Cao T, Zhang W, Wang Q, Wang C, Ma W, Zhang C, et al. Cancer SLC6A6-mediated taurine uptake transactivates immune checkpoint genes and induces exhaustion in CD8(+) T cells. Cell. 2024;187:2288–304.e27. 38565142 10.1016/j.cell.2024.03.011

[26] Lu Z, Bae E-A, Verginadis II, Zhang H, Cho C, McBrearty N, et al. Induction of the activating transcription factor-4 in the intratumoral CD8+ T cells sustains their viability and anti-tumor activities. Cancer Immunol Immunother CII. 2023;72:815–26. 36063172 10.1007/s00262-022-03286-2 PMC10317204

[27] Rashidi A, Miska J, Lee-Chang C, Kanojia D, Panek WK, Lopez-Rosas A, et al. GCN2 is essential for CD8(+) T-cell survival and function in murine models of malignant glioma. Cancer Immunol Immunother. 2020;69:81–94. 31844909 10.1007/s00262-019-02441-6 PMC6952559

[28] Chaveroux C, Bruhat A, Carraro V, Jousse C, Averous J, Maurin A-C, et al. Regulating the expression of therapeutic transgenes by controlled intake of dietary essential amino acids. Nature Biotechnol. 2016;34:746–51. 27272383 10.1038/nbt.3582

[29] Lynn RC, Weber EW, Sotillo E, Gennert D, Xu P, Good Z, et al. c-Jun overexpression in CAR T cells induces exhaustion resistance. Nature. 2019;576:293–300. 31802004 10.1038/s41586-019-1805-z PMC6944329

[30] Doan AE, Mueller KP, Chen AY, Rouin GT, Chen Y, Daniel B, et al. FOXO1 is a master regulator of memory programming in CAR T cells. Nature. 2024;629:211–8. 38600391 10.1038/s41586-024-07300-8 PMC11062920

[31] Yu M, Zhang S. Influenced tumor microenvironment and tumor immunity by amino acids. Front Immunol. 2023;14:1118448. 36798123 10.3389/fimmu.2023.1118448 PMC9927402

[32] Sullivan MR, Danai LV, Lewis CA, Chan SH, Gui DY, Kunchok T, et al. Quantification of microenvironmental metabolites in murine cancers reveals determinants of tumor nutrient availability. eLife. 2019;8:e44235. 30990168 10.7554/eLife.44235 PMC6510537

[33] Yang S, Liu F, Wang QJ, Rosenberg SA, Morgan RA. The shedding of CD62L (L-selectin) regulates the acquisition of lytic activity in human tumor reactive T lymphocytes. PLoS ONE. 2011;6:e22560. 21829468 10.1371/journal.pone.0022560 PMC3145643

[34] Koyama-Nasu R, Wang Y, Hasegawa I, Endo Y, Nakayama T, Kimura MY. The cellular and molecular basis of CD69 function in anti-tumor immunity. Int Immunol. 2022;34:555–61. 35689672 10.1093/intimm/dxac024

[35] Beltra J-C, Manne S, Abdel-Hakeem MS, Kurachi M, Giles JR, Chen Z, et al. Developmental Relationships of Four Exhausted CD8+ T-Cell subsets reveals underlying transcriptional and epigenetic landscape control mechanisms. Immunity. 2020;52:825–41.e8. 32396847 10.1016/j.immuni.2020.04.014 PMC8360766

[36] Maimela NR, Liu S, Zhang Y. Fates of CD8+ T cells in Tumor Microenvironment. Comput Struct Biotechnol J. 2019;17:1–13. 30581539 10.1016/j.csbj.2018.11.004 PMC6297055

[37] Kirsh SM, Pascetta SA, Uniacke J. Spheroids as a 3D Model of the Hypoxic Tumor Microenvironment. In: Ursini-Siegel J, editor. *The Tumor Microenvironment*. 2614. New York, NY: Springer US; 2023. p. 273–85. 10.1007/978-1-0716-2914-7_17 36587131

[38] Lagies S, Schlimpert M, Neumann S, Wäldin A, Kammerer B, Borner C, et al. Cells grown in three-dimensional spheroids mirror in vivo metabolic response of epithelial cells. Commun Biol. 2020;3:246. 32427948 10.1038/s42003-020-0973-6 PMC7237469

[39] Ghanbari Movahed Z, Matin MM, Mansouri K, Sisakhtnezhad S. Amino acid profile changes during enrichment of spheroid cells with cancer stem cell properties in MCF -7 and MDA-MB -231 cell lines. Cancer Rep. 2023;6:e1809. 10.1002/cnr2.1809 PMC10172158 37092500

[40] Wang N, Zeng G-Z, Yin J-L, Bian Z-X. Artesunate activates the ATF4-CHOP-CHAC1 pathway and affects ferroptosis in Burkitt’s Lymphoma. Biochem Biophys Res Commun. 2019;519:533–9. 31537387 10.1016/j.bbrc.2019.09.023

[41] Van Trimpont M, Peeters E, De Visser Y, Schalk AM, Mondelaers V, De Moerloose B, et al. Novel Insights on the Use of L-Asparaginase as an Efficient and Safe Anti-Cancer Therapy. Cancers. 2022;14:902. 35205650 10.3390/cancers14040902 PMC8870365

[42] Blachier J, Cleret A, Guerin N, Gil C, Fanjat J-M, Tavernier F, et al. L-asparaginase anti-tumor activity in pancreatic cancer is dependent on its glutaminase activity and resistance is mediated by glutamine synthetase. Exp Cell Res. 2023;426:113568. 36967104 10.1016/j.yexcr.2023.113568

[43] Weber EW, Parker KR, Sotillo E, Lynn RC, Anbunathan H, Lattin J, et al. Transient rest restores functionality in exhausted CAR-T cells through epigenetic remodeling. Science. 2021;372:eaba1786. 33795428 10.1126/science.aba1786 PMC8049103

[44] Arcangeli S, Bove C, Mezzanotte C, Camisa B, Falcone L, Manfredi F, et al. CAR T-cell manufacturing from naive/stem memory T lymphocytes enhances antitumor responses while curtailing cytokine release syndrome. J Clin Investig. 2022;132:e150807. 35503659 10.1172/JCI150807 PMC9197529

[45] Meyran D, Terry RL, Zhu JJ, Haber M, Ziegler DS, Ekert PG, et al. Early-phenotype CAR-T cells for the treatment of pediatric cancers. Annals Oncol. 2021;32:1366–80. 10.1016/j.annonc.2021.07.018 34375680

[46] Chen T, Wang M, Chen Y, Liu Y. Current challenges and therapeutic advances of CAR-T-cell therapy for solid tumors. Cancer Cell Int. 2024;24:133. 38622705 10.1186/s12935-024-03315-3 PMC11017638

[47] Gomez-Melero S, Hassouneh F, Vallejo-Bermudez IM, Aguera-Morales E, Solana R, Caballero-Villarraso J. Tandem CAR-T-cell therapy: recent advances and current challenges. Front Immunol. 2025;16:1546172. 40092990 10.3389/fimmu.2025.1546172 PMC11907001

[48] Zheng Y, Yao Y, Ge T, Ge S, Jia R, Song X, et al. Amino acid metabolism reprogramming: shedding new light on T-cell anti-tumor immunity. J Exp Clin Cancer Res. 2023;42:291. 37924140 10.1186/s13046-023-02845-4 PMC10623764

[49] Bian Y, Li W, Kremer DM, Sajjakulnukit P, Li S, Crespo J, et al. Cancer SLC43A2 alters T-cell methionine metabolism and histone methylation. Nature. 2020;585:277–82. 32879489 10.1038/s41586-020-2682-1 PMC7486248

[50] Wang W, Zou W. Amino acids and their transporters in T-cell immunity and cancer therapy. Mol Cell. 2020;80:384–95. 32997964 10.1016/j.molcel.2020.09.006 PMC7655528

[51] Yang L, Chu Z, Liu M, Zou Q, Li J, Liu Q, et al. Amino acid metabolism in immune cells: essential regulators of the effector functions, and promising opportunities to enhance cancer immunotherapy. J Hematol Oncol. 2023;16:59. 37277776 10.1186/s13045-023-01453-1 PMC10240810

[52] Sullivan MR, Vander Heiden MG. Determinants of nutrient limitation in cancer. Crit Rev Biochem Mol Biol. 2019;54:193–207. 31162937 10.1080/10409238.2019.1611733 PMC6715536

[53] Muir A, Vander Heiden MG. The nutrient environment affects therapy. Science. 2018;360:962–3. 29853672 10.1126/science.aar5986 PMC6368963

[54] Byun JK, Park M, Lee S, Yun JW, Lee J, Kim JS, et al. Inhibition of glutamine utilization synergizes with immune checkpoint inhibitor to promote antitumor immunity. Mol Cell. 2020;80:592–606.e8. 33159855 10.1016/j.molcel.2020.10.015

[55] Pandit M, Kil YS, Ahn JH, Pokhrel RH, Gu Y, Mishra S, et al. Methionine consumption by cancer cells drives a progressive upregulation of PD-1 expression in CD4 T cells. Nat Commun. 2023;14:2593. 37147330 10.1038/s41467-023-38316-9 PMC10162977

[56] Kosti P, Opzoomer JW, Larios-Martinez KI, Henley-Smith R, Scudamore CL, Okesola M, et al. Hypoxia-sensing CAR T cells provide safety and efficacy in treating solid tumors. Cell Rep Med. 2021;2:100227. 33948568 10.1016/j.xcrm.2021.100227 PMC8080111

[57] Chaveroux C, Carraro V, Canaple L, Averous J, Maurin A-C, Jousse C, et al. In vivo imaging of the spatiotemporal activity of the eIF2α-ATF4 signaling pathway: Insights into stress and related disorders. Sci Signal. 2015;8:rs5. 25921292 10.1126/scisignal.aaa0549

[58] Boissonnas A, Fetler L, Zeelenberg IS, Hugues S, Amigorena S. In vivo imaging of cytotoxic T-cell infiltration and elimination of a solid tumor. J Exp Med. 2007;204:345–56. 17261634 10.1084/jem.20061890 PMC2118741

[59] Chen J, Qiu S, Li W, Wang K, Zhang Y, Yang H, et al. Tuning charge density of chimeric antigen receptor optimizes tonic signaling and CAR-T-cell fitness. Cell Res. 2023;33:341–54. 36882513 10.1038/s41422-023-00789-0 PMC10156745

[60] Lukey MJ, Greene KS, Erickson JW, Wilson KF, Cerione RA. The oncogenic transcription factor c-Jun regulates glutaminase expression and sensitizes cells to glutaminase-targeted therapy. Nat Commun. 2016;7:11321. 27089238 10.1038/ncomms11321 PMC4837472

[61] Serna R, Ramrakhiani A, Hernandez JC, Chen CL, Nakagawa C, Machida T, et al. c-JUN inhibits mTORC2 and glucose uptake to promote self-renewal and obesity. iScience. 2022;25:104325. 35601917 10.1016/j.isci.2022.104325 PMC9121277

[62] Zhu P, Liu G, Wang X, Lu J, Zhou Y, Chen S, et al. Transcription factor c-Jun modulates GLUT1 in glycolysis and breast cancer metastasis. BMC Cancer. 2022;22:1283. 36476606 10.1186/s12885-022-10393-x PMC9730598

[63] Wu H, Zhao X, Hochrein SM, Eckstein M, Gubert GF, Knopper K, et al. Mitochondrial dysfunction promotes the transition of precursor to terminally exhausted T cells through HIF-1alpha-mediated glycolytic reprogramming. Nat Commun. 2023;14:6858. 37891230 10.1038/s41467-023-42634-3 PMC10611730

[64] Zhu X, Chen J, Li W, Xu Y, Shan J, Hong J, et al. Hypoxia-Responsive CAR-T cells exhibit reduced exhaustion and enhanced efficacy in solid tumors. Cancer Res. 2024;84:84–100. 37874330 10.1158/0008-5472.CAN-23-1038

[65] Greenshpan Y, Sharabi O, Ottolenghi A, Cahana A, Kundu K, Kundu K, et al. Synthetic promoters to induce immune-effectors into the tumor microenvironment. Commun Biol. 2021;4:143. 33514819 10.1038/s42003-021-01664-7 PMC7846768

[66] Zennou V, Serguera C, Sarkis C, Colin P, Perret E, Mallet J, et al. The HIV-1 DNA flap stimulates HIV vector-mediated cell transduction in the brain. Nature Biotechnol. 2001;19:446–50. 11329014 10.1038/88115

