# Supplementary material for: Harnessing nutrient scarcity for enhanced CAR-T-cell potency and safety in solid tumors
Source: Cell Mol Immunol. 2025 May 8;22(6):645–60. doi: 10.1038/s41423-025-01290-x (PMC12125372; doi:10.1038/s41423-025-01290-x)
Supplement: Supplementary file 1 — Supplementary Materials [file 41423_2025_1290_MOESM1_ESM.pdf]

Supplemental Information

Harnessing Nutrients Scarcity for Enhanced CAR-T Cell Potency and Safety in Solid Tumours

Enzo Manchon[1], Nell Hirt[1], Benjamin Versier[2], Aravindhan Soundiramourty[2], Ludmila Juricek[2†], Celeste Lebbe[1,3], Maxime Battistella[1,4], Yves Christen[2], Jacques Mallet[2], Dominique Charron[1,2], Nabila Jabrane-Ferrat[5], Che Serguera[2,6*], Reem Al-Daccak[1*#]

Supplementary Figures 1 to 8
Supplementary Tables 1 to 5

[*] These authors equally contributed and jointly supervised this work.

# Corresponding author:

Reem Al-Daccak, PhD

Hôpital Saint-Louis, Inserm U976

1, Avenue Claude Vellefaux, 75010, Paris - France

email: reem.al-daccak@inserm.fr

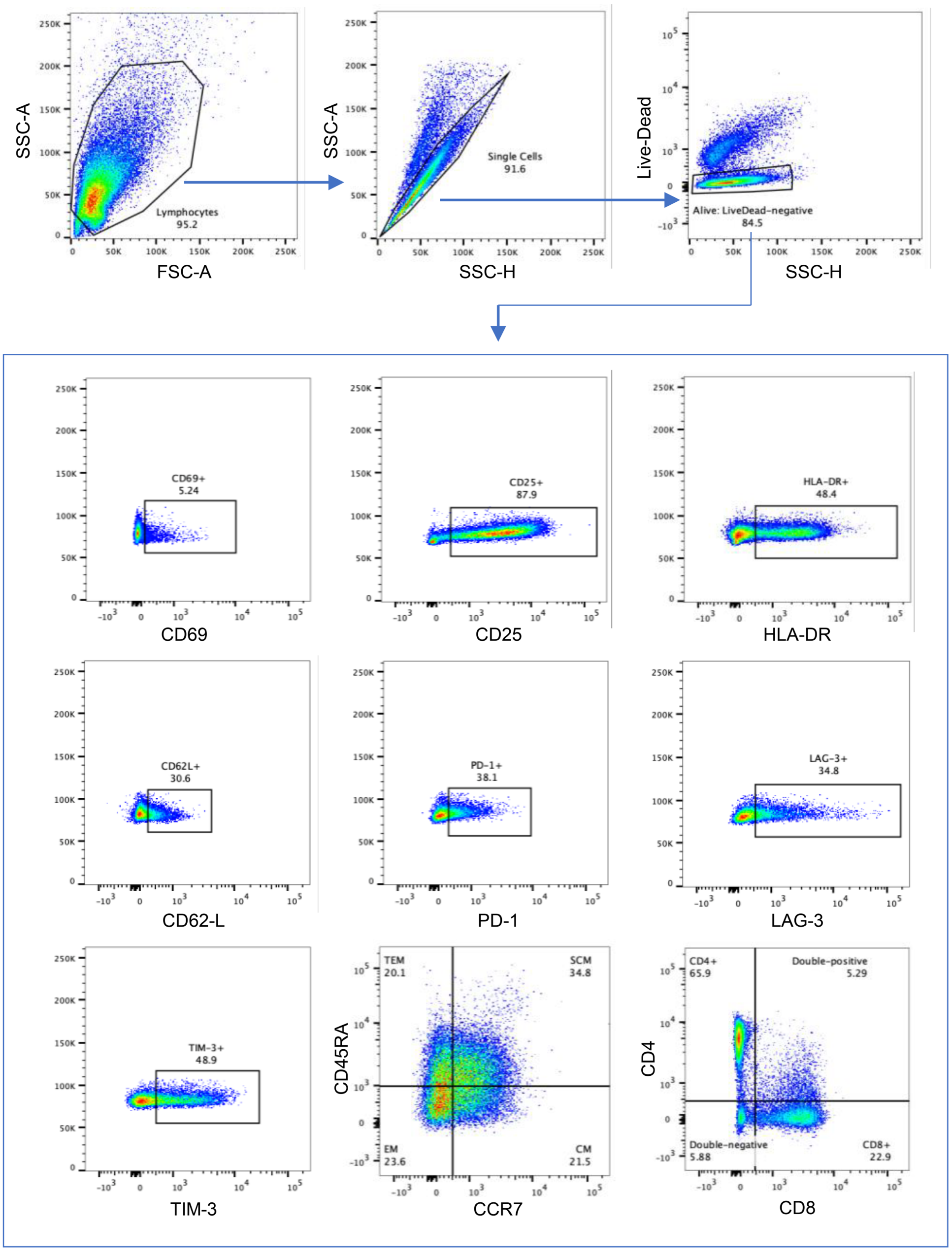

Fig. S1. Gating strategy of T cell phenotypes. The figure shows the gating strategy applied to determine the phenotype of T cells after 48h of culture under amino acid (AA) restriction.

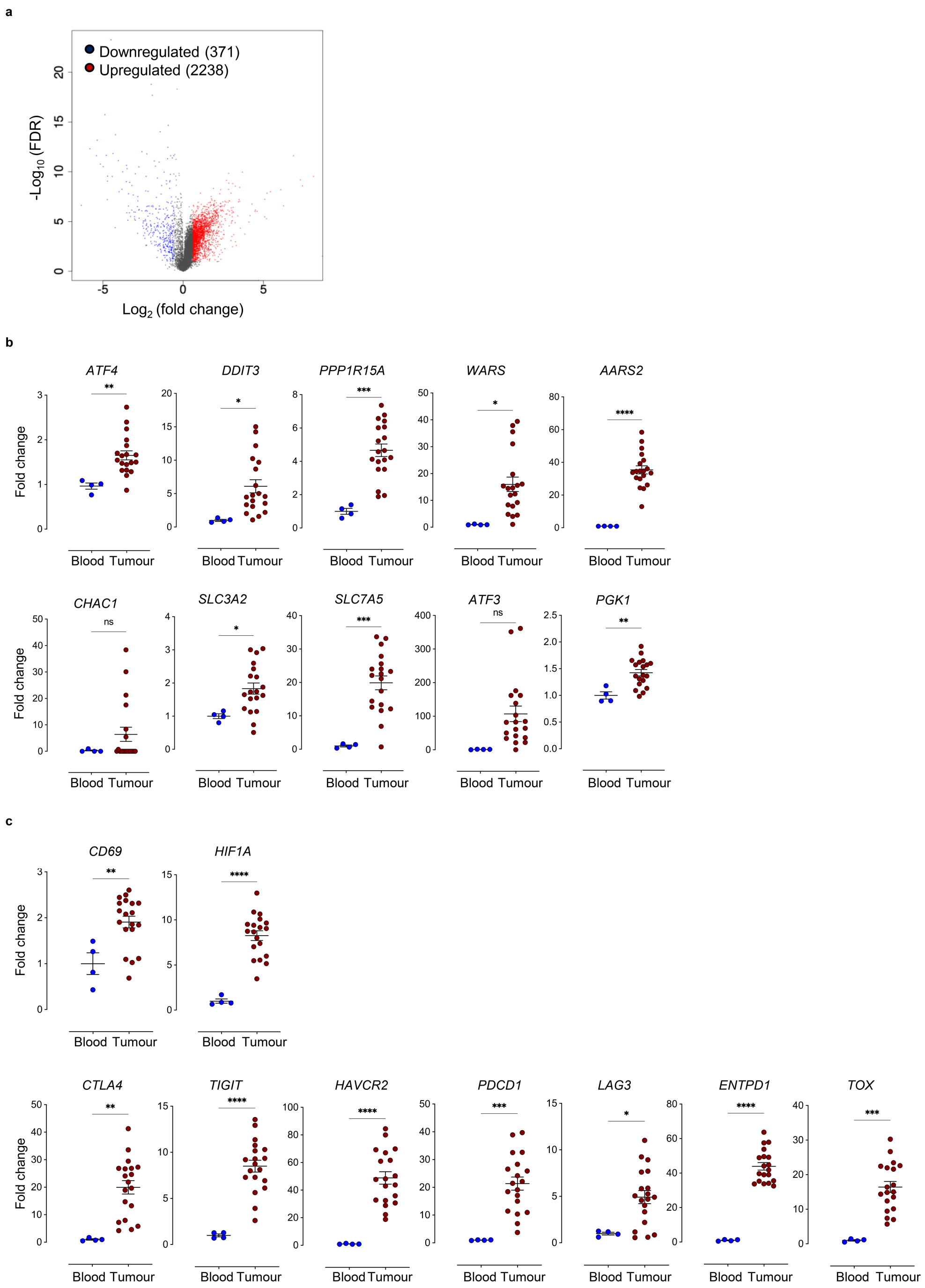

Fig. S2. Differentially expressed genes in melanoma TILs. a Volcano plot presenting differentially expressed genes in melanoma-TILs compared to blood T cells. The $\log_2$FC indicates the fold change of mean gene expression with 1.5 as a threshold. The Y-axis shows the -Log10(FDR) with 0.05 as a threshold. **b,c** Dot plots illustrating the expression (Log_2(TPM/10+1)) of ATF4 and ATF4-target genes mRNA (B) and hypoxia and exhaustion-related mRNA (C) in melanoma-TILs in comparison to blood T cells. Results are fold change gene expression compared to blood T cells. They are representative of 19 melanoma TILs samples compared to 4 healthy donor blood samples, and are presented as mean values ± SEM. Statistical analyses were performed with student t-test. Asterisks represent significant differences between TILs and blood T cells (*p<0.05, **p<0.01, ***p<0.001, ****p<0.0001).

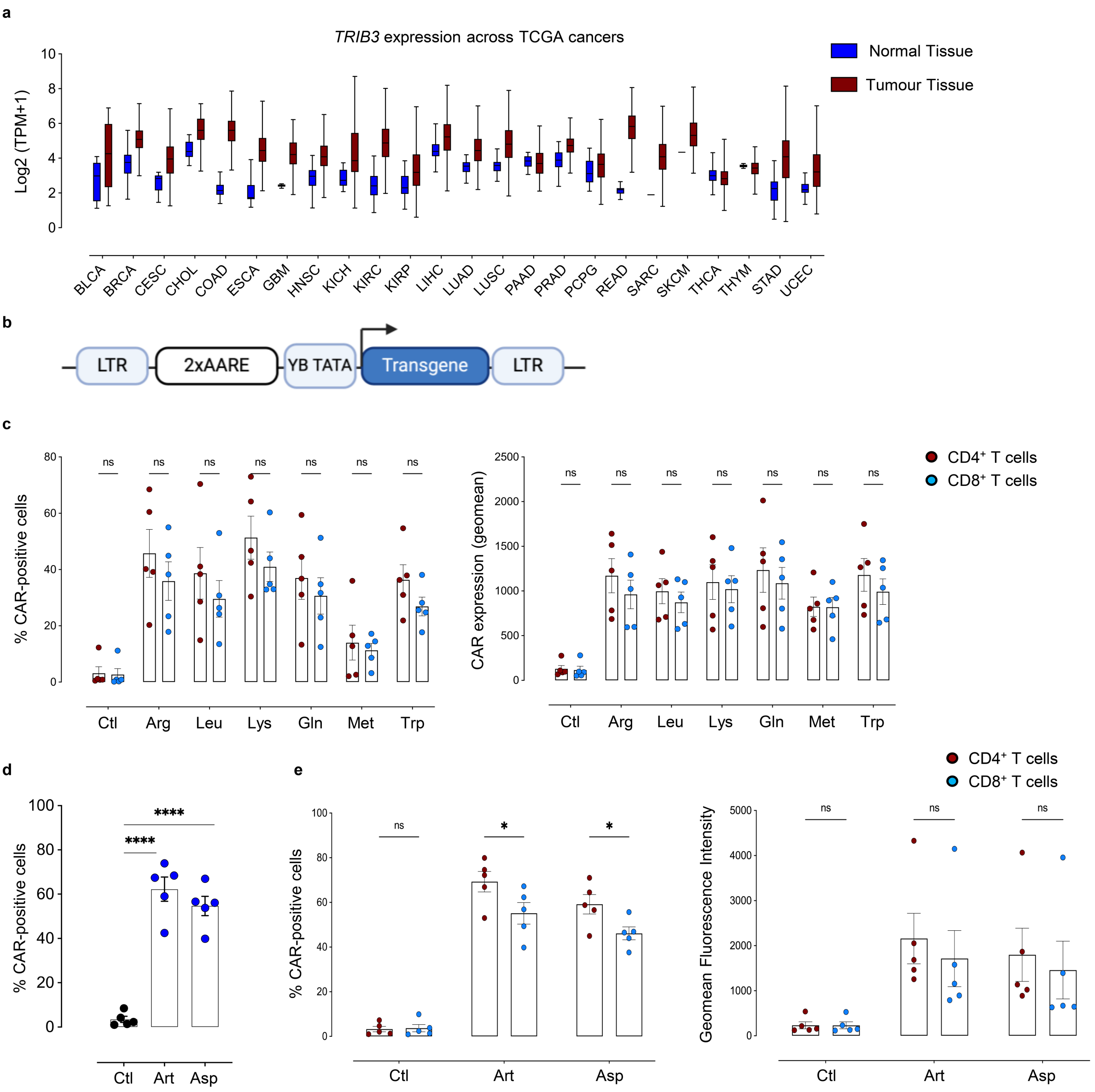

Fig. S3. CAR expression under the control of 2xAARE-YB. a Box plot illustrating the expression (Log_2(TPM+1)) of *Trib3* mRNA across various cancers and their relative healthy tissue controls. **b** Schematic representation of the 2xAARE-YB system. **c** Bar plots showing the percentage of (%) CAR-positive $CD4^+$ and $CD8^+$ 2xAARE-YB-CAR-T cells under AA restrictions. **d,e** Bar plots demonstrating the percentage of (%) of CAR-positive $CD3^+$ (D) and the percentage of (%) and intensity (geometric mean) of CAR-positive $CD4^+$ and $CD8^+$ (E) 2xAARE-YB-CAR-T cells upon exposure to DMSO (Ctl), 1uM Artesunate (Art) or 5U/ml L-asparaginase (Asp). All results are presented as mean values ± SEM of five independent healthy donors. Statistical analyses were performed with one-way Anova. Asterisks represent significant differences between groups (*p<0.05, ****p<0.0001).

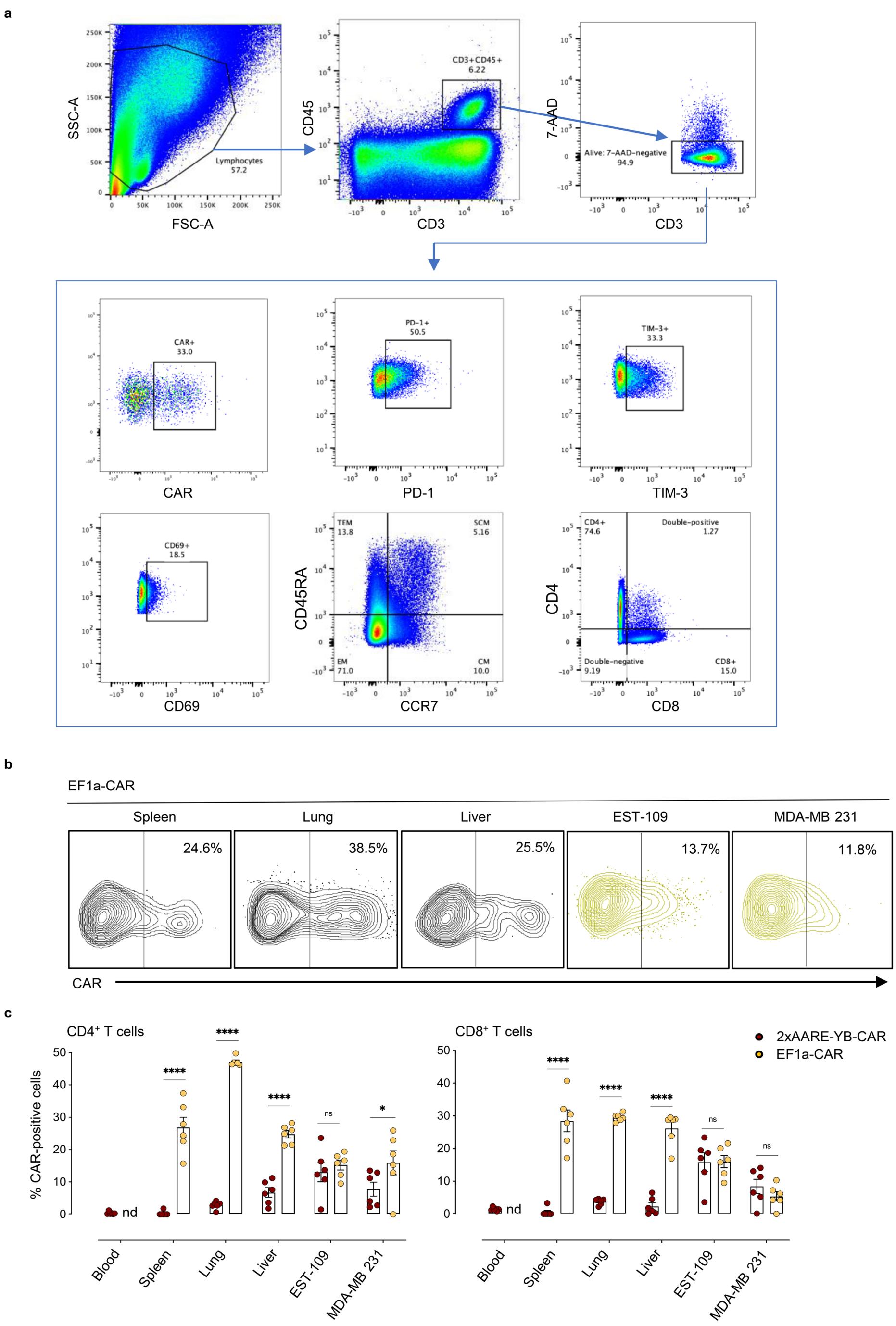

Fig. S4. CAR expression in T cells infiltrating tumours. a Gating strategy applied for phenotyping T cells from tumours isolated from CD19^{+} EST-109 tumour-bearing NXG mice that received *i.t.* injection of either 2xAARE-YB-CAR or EF1a-CAR-T cells. **b** Representative contour plots presenting the percentage of (%) CAR-positive EF1a-CAR-T cells in the blood, spleen, lungs, liver, and CD19^{+}-EST-109 and CD19^{+}-MDA-MB 231 tumours. **c** Bar plot presenting the percentage of (%) CD4^{+} and CD8^{+} CAR-positive 2xAARE-YB-CAR- and EF1a-CAR-T cells in the blood, spleen, lungs, liver, and CD19^{+}-EST-109 and CD19^{+}-MDA-MB 231 tumours. All results are mean values ± SEM obtained with 6 mice/group. Statistical analyses were performed with one- and two-way Anova. Asterisks represent the significant differences between groups (*p<0.05, ****p<0.0001).

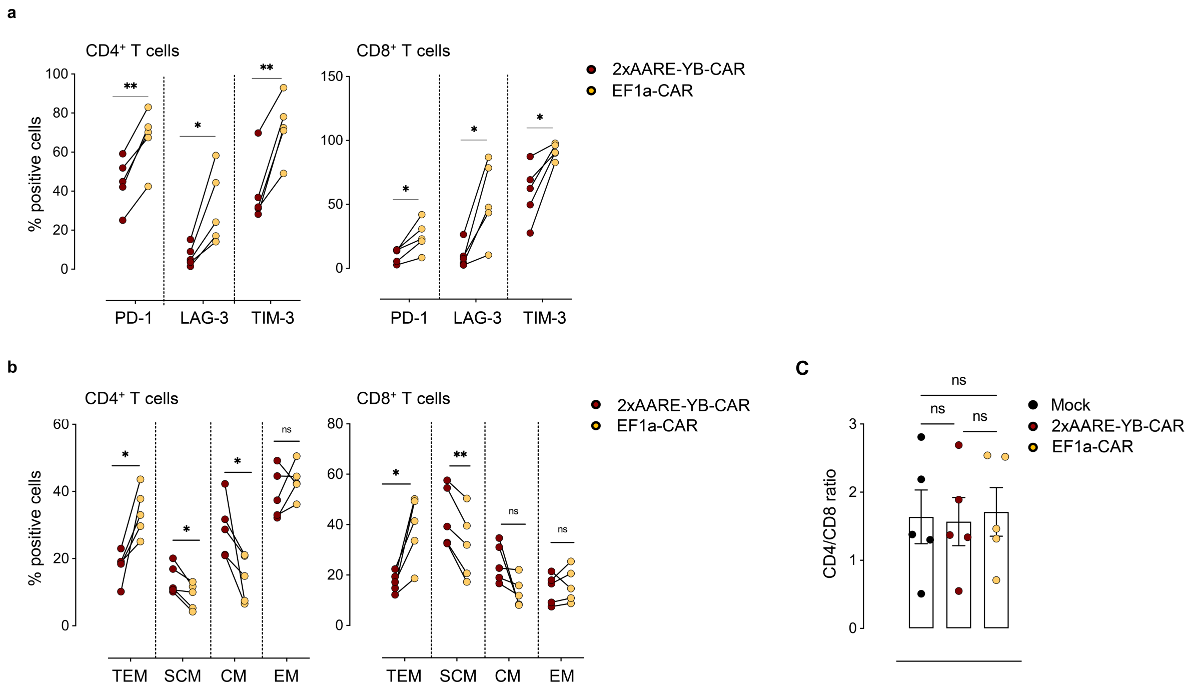

Fig. S5. Phenotype of expanded CAR-T cells. a Connected dot plot presenting the percentage of (%) expanded CD4$^+$ and CD8$^+$ 2xAARE-YB-CAR- and EF1a-CAR-T cells expressing PD-1, LAG-3 or TIM-3. **b** Connected dot plot representing the percentage of (%) expanded CD4$^+$ and CD8$^+$ SCM, CM, EM, and TEM 2xAARE-YB-CAR or EF1a-CAR-T cell subsets. **c** Bar plot representing the ratio of CD4/CD8 in expanded untransduced (mock), 2xAARE-YB-CAR or EF1a-CAR-T cells. All results are obtained with 5 donors and are presented as mean values ± SEM. Statistical analyses were performed with one or two-way Anova. Asterisks represent significant differences between groups (*p<0.05, **p<0.01).

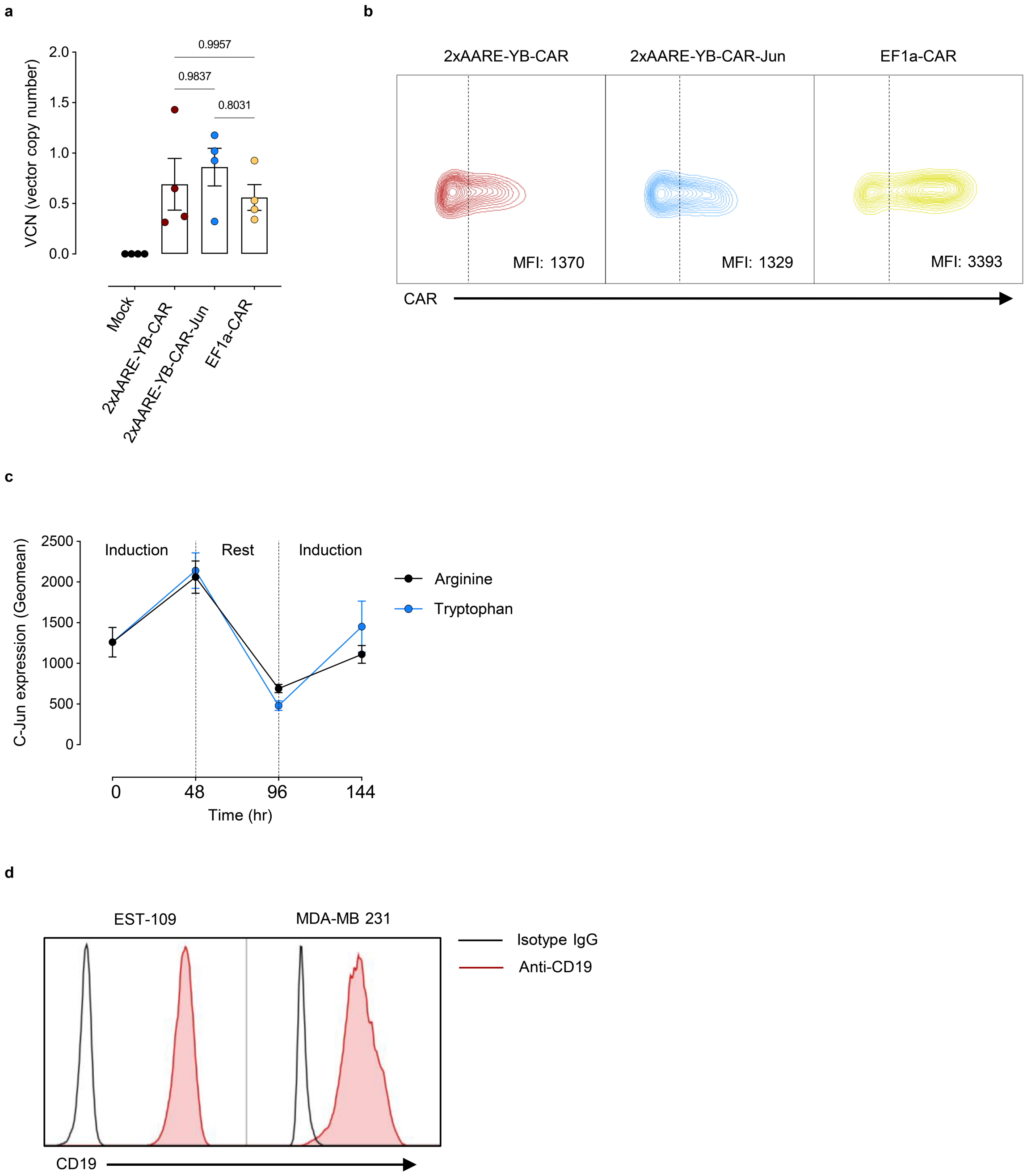

Fig. S6. Co-expression of CAR and c-Jun. a Bar plot showing the number of vector copy integrated per cell (VCN) after transduction of activated T cells with either 2xAARE-YB-CAR, 2xAARE-YB-CAR-Jun or EF1a-CAR vectors. **b** Representative contour plot of CAR expression (MFI; geometric mean) in 2xAARE-YB-CAR-, 2xAARE-YB-CAR-Jun- and EF1a-CAR-T cells. **c** c-Jun expression (geometric mean) in 2xAARE-YB-CAR-Jun-T cells at the indicated times under AA starvation, exposure to control conditions, and re-exposure to AA-restricted conditions. All results are presented as mean values ± SEM obtained with 4 independent healthy donors. Statistical analyses were performed with one- and two-way Anova. Asterisks represent significant differences between groups (*p<0.05, **p<0.01, ****p<0.0001). **d** Representative histogram illustrating CD19 expression in CD19-positive EST-109 and MDA-MB 231 cell lines. Isotype IgG (black empty histogram), anti-CD19 (red filled histogram).

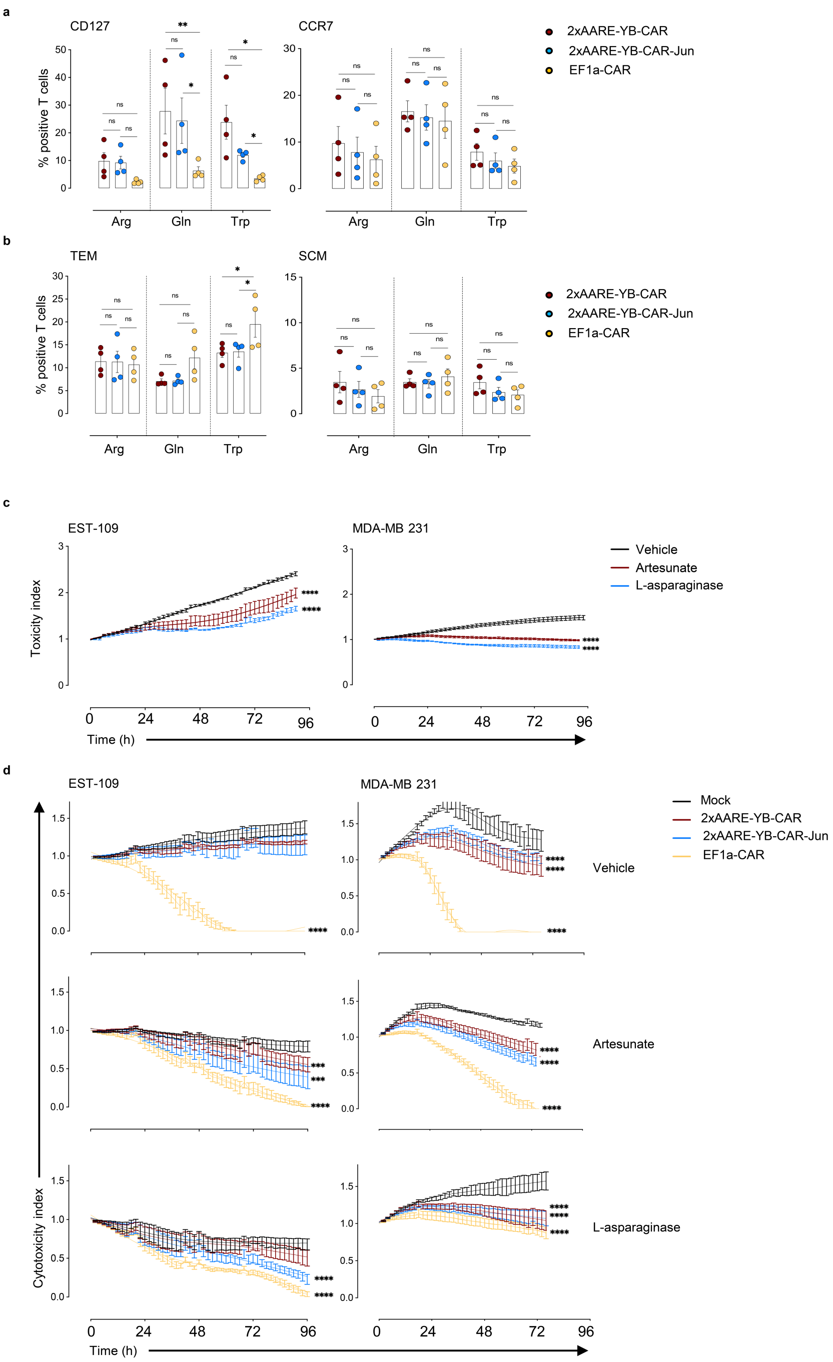

Fig. S7. Phenotype and cytotoxic activity of CAR-T cells in 3D model. a Bar plots representing the percentage of (%) CD127- and CCR7-positive 2xAARE-YB-CAR-, 2xAARE-YB-CAR-Jun- and EF1a-CAR-T cells infiltrating $CD19^{+}$-EST-109 spheroids in AA-restricted conditions. **b** Bar plots representing the percentage of (%) TEM and SCM 2xAARE-YB-CAR-, 2xAARE-YB-CAR-Jun- and EF1a-CAR-T cells infiltrating $CD19^{+}$-EST-109 spheroids in AA-restricted conditions. Asterisks represent significant differences between groups (*p<0.05, **p<0.01). **c** $CD19^{+}$-EST-109 and $CD19^{+}$-MDA-MB 231 spheroids growth curves cultured with DMSO (Vehicle), 1uM Artesunate or 5U/ml L-asparaginase. **d** Cytotoxic activity of untransduced (mock), 2xAARE-YB-CAR-, 2xAARE-YB-CAR-Jun- and EF1a-CAR-T cells against $CD19^{+}$-EST-109 and $CD19^{+}$-MDA-MB 231 spheroids cultured with DMSO (vehicle), 1uM Artesunate or 5U/ml L-asparaginase. All results are presented as mean values ± SEM obtained with 4 independent healthy donors. Asterisks represent significant differences between CAR groups and mock T cells (***p<0.001, ****p<0.0001).

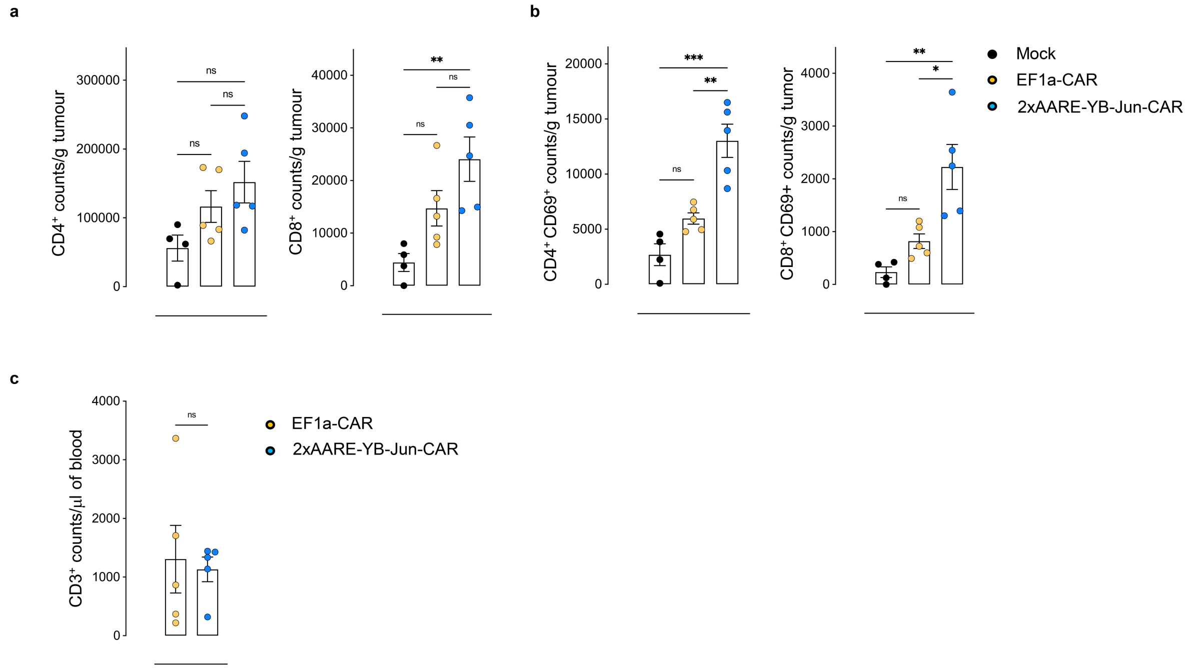

Fig. S8. Phenotype of blood and tumour-infiltrating CAR-T cells. a Bar plot illustrating the absolute count of CD4^{+} and CD8^{+} mock, 2xAARE-YB-CAR-Jun- and EF1a-CAR-T cells per gram of tumour. **b** Bar plot illustrating the absolute count of CD69-positive CD4^{+} and CD8^{+} mock, 2xAARE-YB-CAR-Jun- and EF1a-CAR-T cells per gram of tumour. **c** Bar plot showing the absolute count of mock, 2xAARE-YB-CAR-Jun- and EF1a-CAR-T cells per µl of blood. All results are presented as mean values ± SEM obtained with 5 mice/group. Statistical analyses were performed with student t-test, one- and two-way Anova. Asterisks represent significant differences between groups (*p<0.05, **p<0.01, ****p<0.0001).

Supplementary Table 1: List of key resources

Reagent or Resources	Source	Catalogue number
Antibodies		
BUV805 Mouse Anti-Human CD3 (clone UCHT1)	BD biosciences	Cat# 612895
BUV395 Mouse Anti-Human CD8 (Clone HIT8a)	BD biosciences	Cat# 740303
APC-H7 Anti-Human CD4 (Clone RPA-T4)	BD biosciences	Cat# 560158
CD4 Antibody, anti-human, Vio Bright V600 REAfinity (clone REA623)	Miltenyi Biotec	Cat# 130-129-579
BV786 Mouse Anti-Human CD45RA (Clone HI100)	BD biosciences	Cat# 563870
Alexa Fluor 647 Mouse Anti Human CD197 (CCR7) (clone 150503)	BD biosciences	Cat# 560816
BV421 Mouse Anti-Human CD62L (clone DREG-56)	BD biosciences	Cat# 563862
BD Horizon BV650 Mouse Anti-Human CD127 (clone 5HIL-7R-M21)	BD biosciences	Cat# 563225
CD279 (PD1) Antibody, anti-human, Vio Bright FITC, REAfinity (clone REA1165)	Miltenyi Biotec	Cat# 130-117-681
PE Mouse Anti-Human LAG-3 (CD223) (clone T47-530)	BD biosciences	Cat# 565617
CD366 (TIM-3) Antibody, anti-human, APC, REAfinity (clone REA635)	Miltenyi Biotec	Cat# 130-119-781
CD69 Antibody, anti-human, Vioblue, REAfinity (clone REA824)	Miltenyi Biotec	Cat# 130-112-610
PE Mouse Anti-Human CD25 (clone M-A251)	BD biosciences	Cat# 555432
PE Mouse Anti-Human HLA-DR (clone L243)	BD biosciences	Cat# 560943
CD45 Antibody, anti-human, PE, REAfinity (clone REA747)	Miltenyi Biotec	Cat# 130-110-632
BD Horizon BV650 Mouse Anti-Human CD19 (clone SJ25C1)	BD biosciences	Cat# 563226
Biotin antibody, PE-Vio 770, REAfinity (clone REA746)	Miltenyi Biotec	Cat# 130-110-953
R-Phycoerythrin Streptavidin	Jackson Lab	Cat# 016-110-084
c-Jun (60A8) Rabbit mAb (Alexa Fluor 647 Conjugate)	Cell Signaling	Cat# 40502
Alexa Fluor 647 Mouse IgG1k Isotype Control	BD biosciences	Cat# 557732
BV650 Mouse IgG1k Isotype Control	BD biosciences	Cat# 563231
OneComp eBeads	Invitrogen	Cat# 01-1111-41
ATF-4 (D4B8) Rabbit mAb	Cell Signaling	Cat# 11815
Anti-GAPDH antibody	Abcam	Cat# ab8245
Anti-CD3 antibody (CD3-12)	Abcam	Cat# ab11089
Anti-ATF-4 antibody	Abcam	Cat# ab31390
Secondary antibody (Opal Polymer)	Akoya Biosciences	Cat# ARH1001EA
Biological samples		
Human blood	Healthy donors	Etablissement Français du sang (EFS)
Experimental models: Cell lines		

ESTDAB-109	Dr. Federico Garrido Torres-Puchol (Granada University)	N/A
MDA-MB-231	ATCC	Cat# HTB-26
Experimental models: Organisms/strains		
NXG mice (NOD-*Prkdc*^{scid}-*IL2rg*^{Tm1}/Rj)	Janvier labs	Cat# SM-NXG-M
Critical commercial assays		
CD19 CAR Detection Reagent, human, biotin	Miltenyi Biotec	Cat# 130-129-550
CellTrace CFSE Proliferation kit, for flow cytometry	Thermo Fisher	Cat# C34554
Pan T Cell Isolation Kit, human	Miltenyi Biotec	Cat# 130-096-535
CD19 CAR Detection Reagent, Human	Miltenyi Biotec	Cat# 130-129-550
Tumour Dissociation Kit, Human	Miltenyi Biotec	Cat# 130-095-929
RNeasy Plus Mini Kit	Qiagen	Cat# 74134
BD Cytofix/Cytoperm Fixation/Permeabilization Kit	BD biosciences	Cat# 554714
Chemicals, peptides and recombinant proteins		
Lymphocyte separation medium (Ficoll)	Eurobio Scientific	Cat# CMSMSL01-0U
Dynabeads Human T activator CD3/CD28	Thermo Fisher	Cat# 11131D
Immunocult Human CD3/CD28/CD2 T Cell Activator	StemCell Technologies	Cat# 10970
Recombinant IL-2	Miltenyi Biotec	Cat# 130-097-743
Artesunate	Merck	Cat# PHR2573
L-Asparaginase	Abcam	Cat# ab277068
Corning Matrigel Basement Membrane Matrix	Corning	Cat# 354234
Collagenase, type IV	Thermo Fisher	Cat# 17104019
DNase I	Thermo Fisher	Cat# 18047019
BD Pharmingen 7-AAD	BD biosciences	Cat# 559925
LIVE/DEAD Fixable Lime (506) Viability Kit	Thermo Fisher	Cat# L34990
Opal 520	Akoya Biosciences	Cat# OP001001
Opal 570	Akoya Biosciences	Cat# OP001003
Recombinant DNA		
VSVg 2xAARE-YB-GFP	This study	N/A
VSVg 2xAARE-YB-CD19-41BBz	This study	N/A
VSVg 2xAARE-YB-CD19-41BBz-P2A-c-Jun	This study	N/A
VSVg EF1a-CD19-41BBz	This study	N/A
VSVg TRAP-CD19	This study	N/A
Software and algorithms		
FACSDiva	BD biosciences	N/A
FlowJo v.10.9	TreeStar Inc.	N/A
Prism 10	Graphpad	N/A
Snapgene	GSL Biotech	N/A
R v4.2.2	The R Foundation	N/A
Fiji	Fiji Downloads	N/A

Supplementary Table 2. CAR sequence

Gene	Sequence
anti-CD19-41BBz CAR	5’ATGGCCTTACCAGTGACCGCCTTGCTCCTGCCGCTGGCCT TGCTGCTCCACGCCGCCAGGCCGGACATCCAGATGACACAG ACTACATCCTCCCTGTCTGCCTCTCTGGGAGACAGAGTCACC ATCAGTTGCAGGGCAAGTCAGGACATTAGTAAATATTTAAATT GGTATCAGCAGAAACCAGATGGAACTGTTAAACTCCTGATCT ACCATACATCAAGATTACACTCAGGAGTCCCATCAAGGTTCA GTGGCAGTGGGTCTGGAACAGATTATTCTCTCACCATTAGCA ACCTGGAGCAAGAAGATATTGCCACTTACTTTTGCCAACAGG GTAATACGCTTCCGTACACGTTCGGAGGGGGGACCAAGCTG GAGATCACAGGTGGCGGTGGCTCGGGCGGTGGTGGGTCGG GTGGCGGCGGATCTGAGGTGAAACTGCAGGAGTCAGGACC TGGCCTGGTGGCGCCCTCACAGAGCCTGTCCGTCACATGCA CTGTCTCAGGGGTCTCATTACCCGACTATGGTGTAAGCTGGA TTCGCCAGCCTCCACGAAAGGGTCTGGAGTGGCTGGGAGTA ATATGGGGTAGTGAAACCACATACTATAATTCAGCTCTCAAAT CCAGACTGACCATCATCAAGGACAACTCCAAGAGCCAAGTTT TCTTAAAAATGAACAGTCTGCAAACTGATGACACAGCCATTTA CTACTGTGCCAAACATTATTACTACGGTGGTAGCTATGCTAT GGACTACTGGGGCCAAGGAACCTCAGTCACCGTCTCCTCAA CCACTACCCCAGCACCGAGGCCACCCACCCCGGCTCCTACC ATCGCCTCCCAGCCTCTGTCCCTGCGTCCGGAGGCATGTAG ACCCGCAGCTGGTGGGGCCGTGCATACCCGGGGTCTTGAC TTCGCCTGCGATATCTACATTTGGGCCCCTCTGGCTGGTACT TGCGGGGTCCTGCTGCTTTCACTCGTGATCACTCTTTACTGT AAGCGCGGTCGGAAGAAGCTGCTGTACATCTTTAAGCAACC CTTCATGAGGCCTGTGCAGACTACTCAAGAGGAGGACGGCT GTTCATGCCGGTTCCCAGAGGAGGAGGAAGGCGGCTGCGA ACTGCGCGTGAAATTCAGCCGCAGCGCAGATGCTCCAGCCT ACAAGCAGGGGCAGAACCAGCTCTACAACGAACTCAATCTT GGTCGGAGAGAGGAGTACGACGTGCTGGACAAGCGGAGAG GACGGGACCCAGAAATGGGCGGGAAGCCGCGCAGAAAGAA TCCCCAAGAGGGCCTGTACAACGAGCTCCAAAAGGATAAGA TGGCAGAAGCCTATAGCGAGATTGGTATGAAAGGGGAACGC AGAAGAGGCAAAGGCCACGACGGACTGTACCAGGGACTCA GCACCGCCACCAAGGACACCTATGACGCTCTTCACATGCAG GCCCTGCCGCCTCGG 3’

Supplementary Table 3. Amino acid concentration in control medium and single amino acid-restricted media

Amino acid	RPMI concentration (uM)	*Amino acid restricted-media* (uM)
L-Arginine	1150	1.150
L-Leucine	380	0.38
L-Lysine	220	0.22
L-Methionine	100	0.1
L-Glutamine	2055	2.055
L-Tryptophan	25	0.025

Supplementary Table 4. Sequences of primers used for quantifying mRNA expression in CD3$^+$ T cells using RT-qPCR.

**Gene**	**Sequences**
ASNS	5’ atcactgtcgggatgtaccc 3’; 5’ cttcaacagagtggcagcaa 3’
CAT1	5’ gccattgtcatctccttcctg 3’; 5’ caaacagagacggcctgatg 3’
TRIB3	5’ aactggcatccttgagctgacaac 3’; 5’ aagggattgtccttggacagaggt 3’
DDIT3	5’ tgccaatgatgtgaccctcaatcc 3’; 5’ ttttgtctactccaagccttcccc 3’
PPP1R15A	5’ tgagactcccctaaaggccagaaa 3’; 5’ agacagccaggaaatggacagtga 3’
WARS	5’ gaaaggcattttcggcttca 3’; 5’ cagcctggatggcagga 3’
RICTOR	5’ agtgaatctgtgccatcgagt 3’; 5’ agtagagctgctgccaaacc 3’
CHAC1	5’ tggattttcgggtacggctc 3’; 5’ acttcagggccttgcttacc 3’
SLC7A1	5’ accttctgcattgtgaccgt 3’; 5’ cagcatccacacagcaaacc 3’
SESN2	5’ acttccgccactcagagaag 3’; 5’ gtcaggtcatgtagcgggtg 3’
PSAT1	5’ gaattgctagctgttccagaca 3’; 5’ tcagcacaccttcctgcttt 3’
PYCR1	5’ tgccttgcatgtgctggagagt 3’; 5’ gcttcaccttgtccaggatggt 3’
PHGDH	5’ acgtgtttacggaagagccg 3’; 5’ cccttcaccatgtccacgaa 3’
AARS	5’ ggaccatcactgtggcact 3’; 5’ cggagaatccgtctcaacac 3’
YARS	5’ aaggactttgctgctgaggt 3’; 5’ tccagcaacttgttcagtgc 3’
CALR	5’ agcagaacatcgactgtggg 3’; 5’ ccacagatgtcgggaccaaa3’
SEL1L	5’ cctgaggcacgatctggatttta 3’; 5’ aataccaagtgggtggagagcttc 3’
EDEM1	5’ gtggctgagcaggaactttaga 3’; 5’ aataggagctggatgctgggaatg 3’
HYOU1	5’ gaagatgcagagcccatttc 3’; 5’ tctgctccaggacctcctaa 3’
HERPUD1	5’ tgagcacagcatggcttgtctt 3’; 5’ agcctccaacagctacagcacaaa 3’
SSR3	5’ gttcatcgtgtctgccatcc 3’; 5’ catcctccctcttctgtgctac 3’
HRD1	5’ cctgtggcatttctgcgtcttact 3’; 5’ caggcattcctttccctgagttgt 3’
HK2	5’ tcccctgccaccagacta 3’; 5’ tggacttgaatcccttggtc 3’
SLC2A1	5’ ggttgtgccatactcatgacc 3’; 5’ cagataggacatccagggtagc 3’
PGK1	5’ ctgtggcttctggcatacct 3’; 5’ cgagtgacagcctcagcata 3’
SLC3A2	5’ gcttccaagaatgctgaggtt 3’; 5’ gcagtcggatcaattgaggt 3’
SLC7A5	5’ ccgtctacttcttcggggtc 3’; 5’ cttctgacacaggacggtcg 3’
NFATC1	5’ tggaccagttgtacctggatg 3’; 5’ gtgctccaatgtggcaacta 3’
NFKB1	5’ cctggaaccacgcctcta 3’; 5’ ggctcatatggtttcccattta 3’
NRF2	5’ gagacaggtgaatttctcccaat 3’; 5’ tttgggaatgtgggcaac 3’
TBX21	5’ gcctaccagaatgccgagatta 3’; 5’ ggactcaaagttctcccggaat 3’
ATF3	5’ cgctggaatcagtcactgtcag 3’; 5’ cttgtttcggcactttgcagctg 3’
FOS1L	5’ ggaggaaggaactgaccgactt 3’; 5’ ctctaggcgctccttctgcttc 3’
CEBPG	5’ gcttacagcaggttcctcagct 3’; 5’ cgttgccgatactcgtcactgt 3’
JUN	5’ ccttgaaagctcagaactcggag 3’; 5’ tgctgcgttagcatgagttggc 3’
BATF3	5’ accgagttgctgctcagagaag 3’; 5’ aggtgcttcagctcctctgtca 3’
FOS	5’ gcctctcttactaccactcacc 3’; 5’ agatggcagtgaccgtgggaat 3’
GUSB	5’ tgcgtagggacaagaaccac 3’; 5’ gggaggggtccaaggatttg 3’

Supplementary Table 5. List of gene associated with exhaustion, ER-stress and the ATF4 pathway.

Exhaustion	ER-stress		ATF4 pathway
HAVCR2	AMFR	INSIG2	APOE
KLRC1	ARMET	MAPK10	ASNS
SRGAP3	ATF4	MAPK8	ATF3
FABP5	ATF6	MAPK9	ATG5
ENTPD1	ATF6B	MBTPS1	BGLAP
CSF1	ATXN3	MBTPS2	CA9
PDCD1	BAX	NPLOC4	CCL2
TNFSF4	CALR	NUCB1	CEBPB
GZMA	CANX	OS9	CHAC1
MKI67	CCT4	PDIA3	CXCL8
LGALS3	CCT7	PFDN2	DDIT3
TOX	CEBPB	PFDN5	DDIT4
TIGIT	CREB3	PPIA	DDR2
FUT8	CREB3L3	PPP1R15A	DISC1
TNFRSF9	DDIT3	PRKCSH	FGF19
IL2RB	DELR1	RNF139	FGF21
LAG3	CHOP	RNF5	HRK
NAB1	DNAJB2	RPN1	HSPA5
SLC2A8	DNAJB9	SCAP	IGFBP1
CTLA4	DNAJC10	SEC62	IL6
ITM2A	DNAJC3	SEC63	IRF7
GCNT1	DNAJC4	SEL1L	MAP1LC38
PSMB8	EIF2	SELS	MCL1
PLSCR1	CASP12	SERP1	NDC80
KCNK5	EIF2A	SIL1	NUPR1
TNIP3	EIF2AK3	SREBF1	PLAU
BUB1	CRELD2	SREBF2	PTGS2
PSMA1	ERN2	SYVN1	S100P
TANK	ERO1L	TCP1	SIGMAR1
SUB1	TRB3	UBXN4	SIRT1
ACOT7	ERP44	UFD1L	SIRT2
NEDD9	FBX06		TNFRSF108
HMGB2	GANAB		TRIB3
SDF2L1	GANC		VEGFA
ETFB	HERPUD1		SLC7A1
EZH2	HSPA1B		PPP1R15A
MCM3	HSPA1L		WARS
MIS18BP1	HSPA2		RICTOR
RAD21	HSPA4		SESN2
PSMD9	HSPA4L		PSAT1
SLC43A3	HSPA5		PYCR1
PMF1	HSPH1		PHGDH
	HTRA2		AARS
	INSIG1		YARS
