# Supplementary figures and images for: Harnessing nutrient scarcity for enhanced CAR-T-cell potency and safety in solid tumors
Source: Cell Mol Immunol. 2025 May 8;22(6):645–60. doi: 10.1038/s41423-025-01290-x (PMC12125372; doi:10.1038/s41423-025-01290-x)

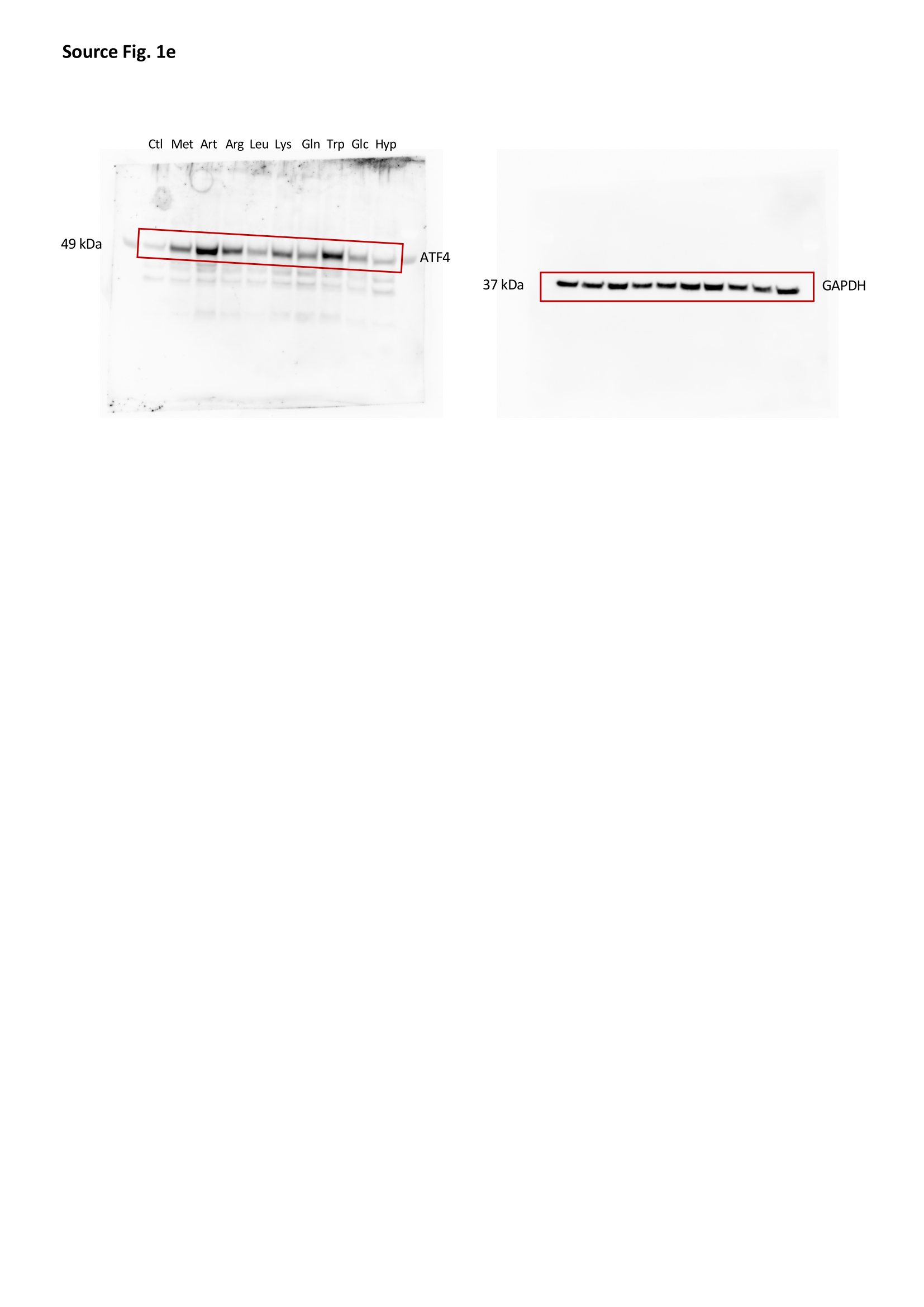

Supplement: Supplementary file 2 — Unprocessed Images [file 41423_2025_1290_MOESM2_ESM.tif]
